# The Ornaments of the Arma Veirana Early Mesolithic Infant Burial

**DOI:** 10.1007/s10816-022-09573-7

**Published:** 2022-08-30

**Authors:** C. Gravel-Miguel, E. Cristiani, J. Hodgkins, C. M. Orr, D. S. Strait, M. Peresani, S. Benazzi, G. Pothier-Bouchard, H. M. Keller, D. Meyer, D. Drohobytsky, S. Talamo, D. Panetta, A. Zupancich, C. E. Miller, F. Negrino, J. Riel-Salvatore

**Affiliations:** 1 Département d’anthropologie, Université de Montréal, Montréal, QC Canada; 2 DANTE – Diet and ANcient TEchnology Laboratory, Department of Oral and Maxillo-Facial Sciences, Sapienza University of Rome, Rome, Italy; 3 Department of Anthropology, University of Colorado Denver, Denver, CO USA; 4 Department of Cell and Developmental Biology, University of Colorado School of Medicine, Aurora, CO USA; 5 Department of Anthropology, Washington University, St. Louis, MO USA; 6 Palaeo-Research Institute, University of Johannesburg, Auckland Park, Johannesburg, South Africa; 7 Prehistory and Antropology Science Unit, Department of Humanities, University of Ferrara, Sezione Di Scienze Preistoriche E Antropologiche, Ferrara, Italy; 8 Institute of Environmental Geology and Geoengineering (IGAG), National Research Council, Milan, Italy; 9 Department of Cultural Heritage, University of Bologna, Ravenna, Italy; 10 Department of Human Evolution, Max Planck Institute for Evolutionary Anthropology, Leipzig, Germany; 11 Department of Anthropology, Yale University, New Haven, CT USA; 12 Cultural Heritage Engineering Initiative (CHEI), University of California San Diego, La Jolla, CA USA; 13 Department of Chemistry G. Ciamician, Alma Mater Studiorum, University of Bologna, Bologna, Italy; 14 Institute of Clinical Physiology - CNR-IFC, Pisa, Italy; 15 Archaeology of Social Dynamics, Institución Milá Y Fontanals, Spanish National Research Council (CSIC), Barcelona, Spain; 16 Institute for Archaeological Sciences and Senckenberg Centre for Human Evolution and Paleoenvironment, University of Tübingen, Tübingen, Germany; 17 SFF Centre for Early Sapiens Behaviour (SapienCE), University of Bergen, Bergen, Norway; 18 Department of Antiquities, Philosophy, History, University of Genoa, Genoa, Italy; 19 Département des sciences historiques, Université Laval, Québec, Canada

**Keywords:** Early Mesolithic, Italy, Infant, Burial, Ornaments, Use-wear analysis

## Abstract

Personal ornaments are widely viewed as indicators of social identity and personhood. Ornaments are ubiquitous from the Late Pleistocene to the Holocene, but they are most often found as isolated objects within archaeological assemblages without direct evidence on how they were displayed. This article presents a detailed record of the ornaments found in direct association with an Early Mesolithic buried female infant discovered in 2017 at the site of Arma Veirana (Liguria, Italy). It uses microscopic, 3D, and positional analyses of the ornaments as well as a preliminary perforation experiment to document how they were perforated, used, and what led to their deposit as part of the infant’s grave goods. This study provides important information on the use of beads in the Early Mesolithic, in general, as well as the relationship between beads and young subadults, in particular. The results of the study suggest that the beads were worn by members of the infant’s community for a considerable period before they were sewn onto a sling, possibly used to keep the infant close to the parents while allowing their mobility, as seen in some modern forager groups. The baby was then likely buried in this sling to avoid reusing the beads that had failed to protect her or simply to create a lasting connection between the deceased infant and her community.

**Supplementary Information:**

The online version contains supplementary material available at 10.1007/s10816-022-09573-7.

## Introduction

### Personal Ornaments in Prehistory

Personal ornaments are widely viewed as indicators of social identity and personhood (*e.g.*, Bar-Yosef Mayer, 2015; Cvitkušić, 2017; Miller, 2009; Newell, 1990; Rigaud, 2011; Stiner, 2014; Vanhaeren & d’Errico, 2011). In modern forager populations, beads compose ornaments that are used to build and maintain social networks (Wiessner, 1982), create, uphold, and showcase people’s selves and social identity (Miller, 2009), and act as protection from evil (*e.g.*, Lévi-Strauss 1995 in Borić & Cristiani, 2019), among other functions. Based on such ethnographic examples, archaeologists have assumed that past ornaments were also created and used for self-embellishment, protection, construction of personhood (*e.g.*, Borić & Cristiani, 2019), and as ethnocultural, age, gender or status markers (*e.g.*, Bar-Yosef Mayer, 2015; Kuhn & Stiner, 2007).

Archaeologically, ornaments are ubiquitous from the Late Pleistocene to the Holocene (Nowell & Cooke, 2021; Sehasseh *et al*., 2021; Stiner, 2014) and recent evidence points toward their autonomous adoption by Neanderthals (*e.g.*, Frayer *et al*., 2020; Moro Abadía & Nowell, 2015; Peresani *et al*., 2013; Romandini *et al*., 2014; Zilhão, 2014). As they provide important information to understand the evolution of modern behavior, social identities, and practices (*e.g.*, Vanhaeren & d’Errico, 2006; Rigaud, 2011; Taborin, 1993), archaeologists have aimed to document the spatial and chronological evolution of their adoption, manufacture, and usage. Unfortunately, as individual beads tend to be small, modular, and portable components of ornaments, they are most often found discarded as isolated objects within archaeological assemblages without clear information on how they were displayed (*e.g.*, as jewelry or sewn on clothes or objects). To get that type of information and understand what ornaments meant to the people who wore them, one has to rely on beads discovered in primary burials (Vanhaeren & d’Errico, 2011, p. 70), as only those contexts can preserve the position in which ornaments were placed on the body.

Primary burials are a privileged setting to study ornaments; unfortunately, burials with body ornaments are relatively rare in the Upper Paleolithic (Riel-Salvatore & Gravel-Miguel, 2013) and the Early Mesolithic (Orschiedt, 2016). The 2013 study performed by part of our team (CGM and JRS) counted 85 burials comprising 117 individuals for the Upper Paleolithic, out of which ~ 48% were adorned with body ornaments (Riel-Salvatore & Gravel-Miguel, 2013). More recent research focusing on the Late Upper Paleolithic and the Early Mesolithic has added a few burials to this list (Orschiedt, 2018). From that revised list of burials dated from the Aurignacian to the Early Mesolithic, ~ 43% of individuals were buried in direct association with body ornaments—the uncertainty in the numbers comes from the difficulty to assign ornaments to specific individuals buried in close quarters within group burials. Out of those, the demographic distribution is as follows: ~ 48 adults, ~ 12 teens, ~ 25 children, and ~ 7 infants including the burial presented here. Therefore, while we have a relatively “good” sample of adorned adults allowing us to identify potential patterns of ornament use, we do not have yet a good understanding of the relationship between infants and ornaments. Moreover, as many Upper Paleolithic burials were excavated a long time ago with excavation methods that did not carefully document the ornaments’ position on the body, reconstructing how beads were worn can prove difficult even when relying on mortuary contexts.

Fortunately, as new burials are discovered and old ones are re-analyzed, new patterns are emerging on the use of ornaments for children and young infants. Some recent articles argue that body ornaments found in certain infant burials were likely attached to a fixed object, possibly a blanket, or baby carrier or sling rather than being worn directly as personal ornaments by the young individuals (Henry-Gambier *et al*., 2019, p. 199; Laporte *et al*., 2021; Laporte & Dupont, 2019; Vang Petersen, 2016). In fact, recent research suggests that the need for baby carriers may have emerged as soon as hominins became bipedal (Langley & Suddendorf, 2020; Suddendorf *et al*., 2020; Taylor, 2010) and that early *Homo* mothers may have carried their infants to the front of their bodies to increase interaction (Nowell & Kurki, 2020). As Vang Petersen argues (2016), baby carriers were more than likely a common occurrence for prehistoric infants and young children due to the need for parents to remain mobile while taking care of their progeny. Recent functional investigations carried out on various sets of ornaments found in burials further support this interpretation, as they suggest that the use of ornaments in association with infants likely produced a sensorial experience beyond the visual (Rainio & Mannermaa, 2014). For example, based on use-wear and experimental analyses, 32 perforated wild boar (*Sus scrofa*) teeth found in the Skateholm burial of a woman buried with her newborn baby (Larsson, 1984, p. 20) were interpreted as rattling ornaments sewn on a baby pouch (Rainio & Tamboer, 2018).

This article aims to add to the growing literature on how ornaments were used for prehistoric infants by presenting a detailed record of the ornaments found in direct association with a female infant buried at Arma Veirana (Liguria, Italy) (AVH-1, nicknamed “Neve,” as per Hodgkins *et al*., 2021). Part of our team recently published on the general characteristics of the burials, including its archaeological setting and the biological profile of the infant (*e.g.*, sex, age at death, and isotopic signature of diet). Here, we present an in-depth analysis focusing on the shell ornaments found in the burial. This research adds detailed information on Early Holocene burial practices and the use of personal ornaments in the past, focusing on the social relationships they fostered among individuals as well as on their possible role(s) in the protection of young children. It then uses ethnographic data to provide possible interpretations of how the beads were used by the Arma Veirana group, and why they found their resting place in the burial.

### The Site

**Fig. 1:**
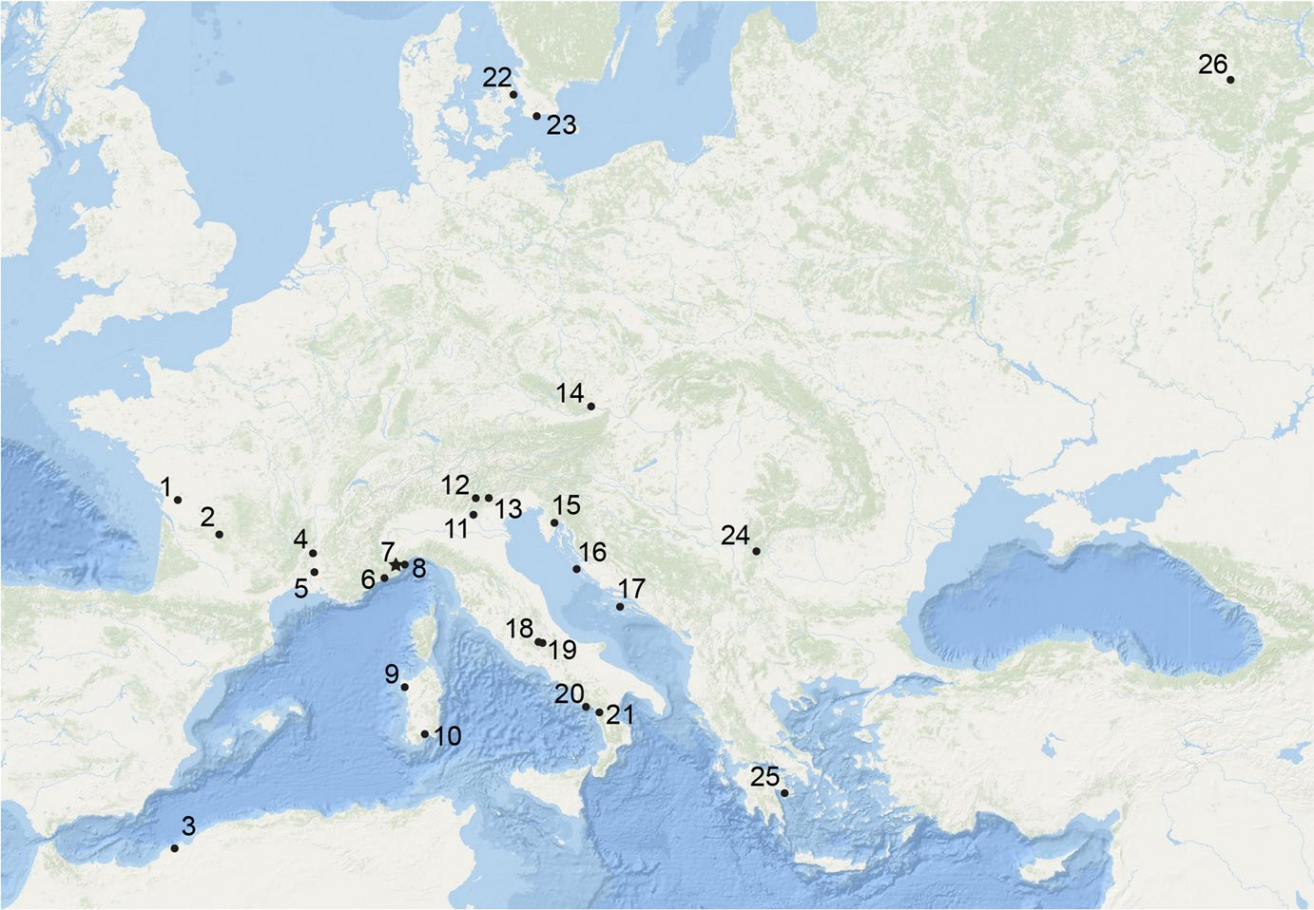
Sites mentioned in the text. Legend: 1, La Vergne; 2, Abri Labattut; 3, Grotte du Polygone; 4, Aven des Iboussières; 5, Avignon-La Balance-Ilot P; 6, Balzi Rossi and Grotta dei Fanciulli (or Grotte des Enfants); 7, Arma Veirana; 8, Caverne delle Arene Candide; 9, Anghelu Ruju; 10, Padru Jossu; 11, Riparo Tagliente; 12, Romagnano Loc III; 13, Riparo Dalmeri; 14, Krems-Wachtberg; 15, Pupicina Cave; 16, Vlakno Cave; 17, Vela Spila; 18, Grotta Continenza; 19, Grotta di Pozzo; 20, Grotta della Serratura; 21, Grotta del Romito; 22, Bogebakken; 23, Skateholm; 24, Vlasac; 25, Franchthi Cave; and 26, Sungir

**Fig. 2:**
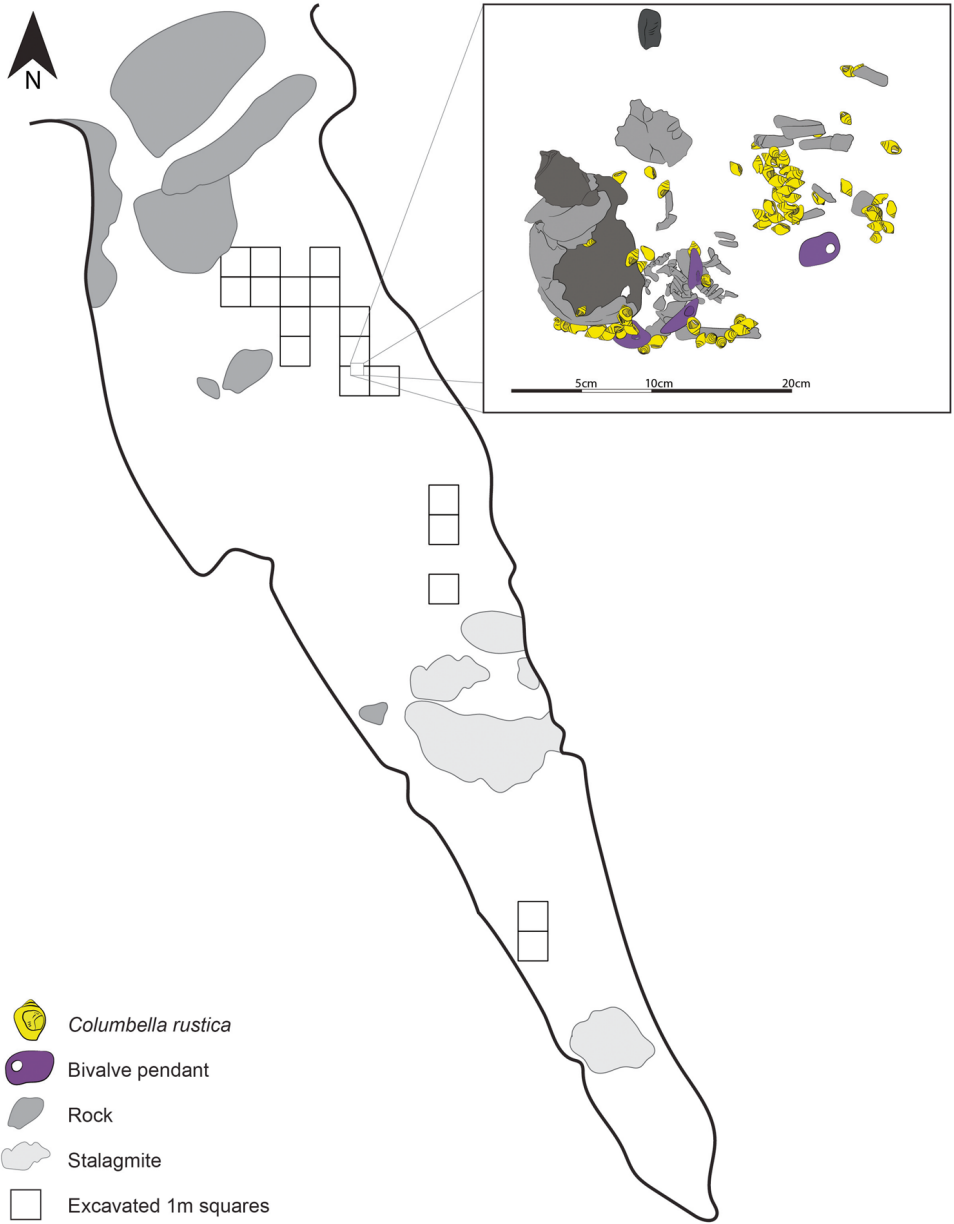
Map of the site and squares excavated. The insert shows the position of the buried infant within the excavation grid

The site of Arma Veirana is located in the Neva Valley that cuts through the Ligurian Prealps and flows into the Centa River valley, which merges with the Mediterranean Sea south of the city of Albenga (Fig. 1). The cave is located ~ 14 km from the current coastline but would have been further inland during its Upper Paleolithic and Early Holocene occupations. It is a ~ 44-m-long triangular chamber opening to the North (Fig. 2).

The site excavation that began in 2015 primarily aimed to document the transition between the Neanderthal and modern human occupations visible in sediments exposed in pits created by unknown looters. However, the excavation also uncovered more recent material, explored in 2017 through the opening of a NS trench located near the Eastern wall of the cave. This is where the burial was discovered.

During excavation of the burial, all excavated sediment was kept for post-excavation processing, including dry sieving, elutriation, and aDNA testing (see Hodgkins *et al*., 2021 for details). Every artifact or bone recovered in or near the remains was carefully freed from the surrounding sediment and photographed in situ by DM and DD using photogrammetry. Photo chits were placed around each artifact and shot in the total station, which allowed georectification of the photogrammetric model of each artifact. This method allowed the team of CISA3 at University of California San Diego to recreate a 3D model of the human remains and artifacts excavated during the two field seasons. This model proved indispensable to understanding the placement of the remains and grave goods, and to reconstruct how the burial took place and how its material shifted post-depositionally. For a more detailed account of the excavation methods used at the site as well as a more detailed history of the burial discovery, see Hodgkins *et al*. (2021).

### Burial Characteristics

Excavation of the burial and its immediate surroundings showed that the infant was buried in a shallow ~ 30 × 15 cm pit located ~ 2 m from the nearest wall and ~ 8.5 m from the nearest current cave opening. The pit was dug within a slowly eroding semi-sterile layer to the north, south, and east. To the west of the burial, the semi-sterile layer was superposed by a layer containing a high density of faunal remains, which were piece plotted using the total station, but have not yet been studied or dated. To date, it is unclear if those are contemporaneous with the burial or not. A sample obtained from one of AVH-1 vertebrae dates to 10,210–9910 cal. BP (95.4% probability, Hodgkins *et al*., 2021), which places the burial in the regional Early Mesolithic. Additional dates obtained from faunal remains and charcoal samples collected from the burial fill fall within two ranges—(1) the Late Epigravettian at ~ 15,500 cal. years BP and (2) the Early Mesolithic at ~ 10,200 cal. years BP. The presence of older bones in the sediment found above the human remains suggests that the pit was dug into older sediment that originally contained them and that was subsequently used to cover the buried infant.

**Fig. 3:**
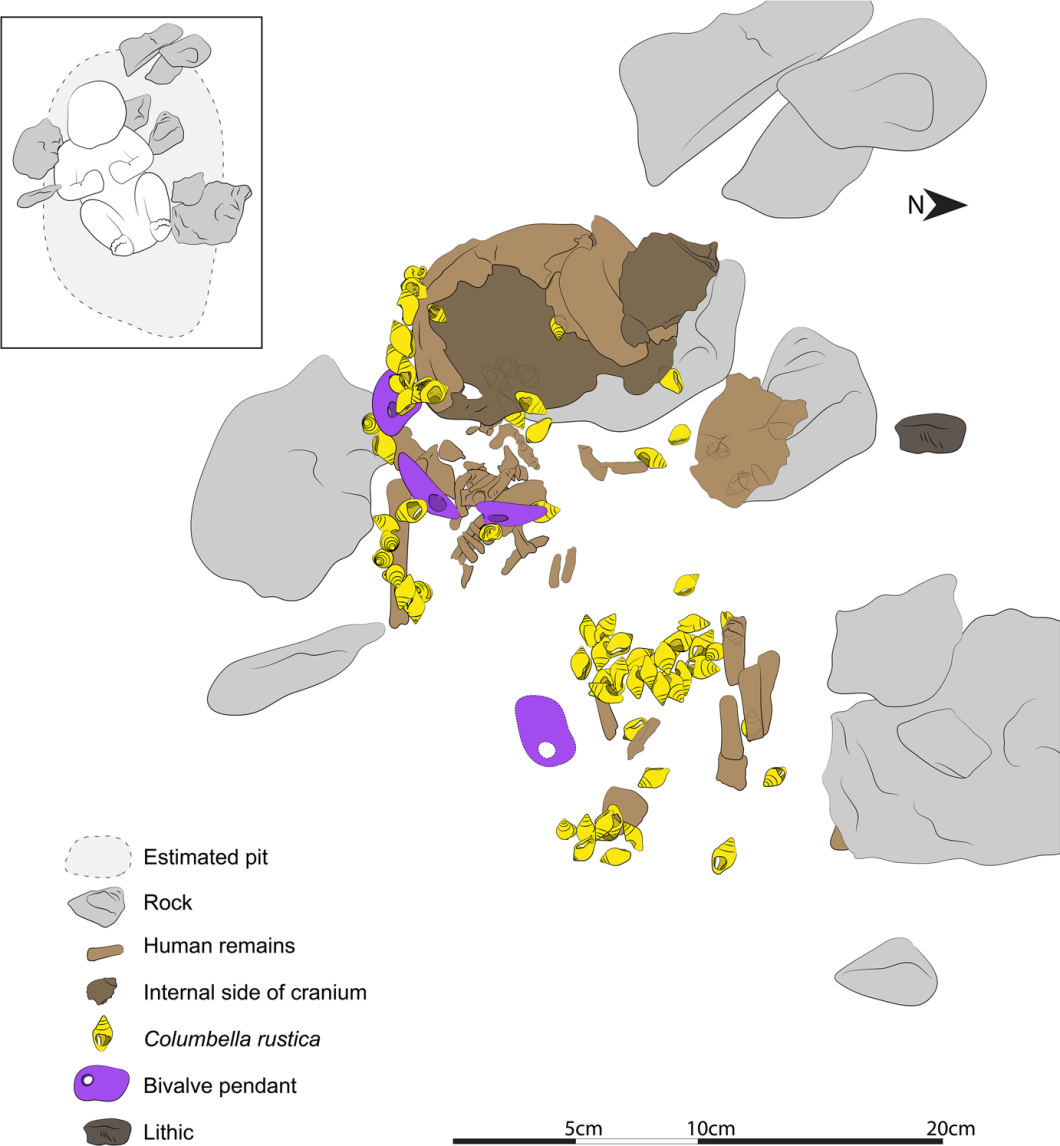
Position of the human remains and associated grave goods. Dotted lines show artifacts found significantly higher or lower than the human remains or that were located under the drawn pieces. This tracing was produced by CGM combining the 3D photogrammetry reconstruction of the burial, field photos, GIS data, and the micro-CT scans of each shell

aDNA and tooth analyses have shown that the infant was a ~ 40- to 50-day-old female (Hodgkins *et al*., 2021). Combined with the 3D reconstruction of the burial, CMO’s study of the remains shows that she was placed in the pit in a supine position oriented along an EW axis with her head toward the west wall. The size of her right humerus (17.5 mm perimeter measured at the middle of the long bone) suggests that the infant would have been ~ 50- to 55-cm tall (as per Dedet *et al*.’s (1991) method), which is ~ 20 cm more than the length of the pit, thus suggesting that the infant was buried with her legs folded back on top of her abdomen, as shown in Fig. 3.

Within the burial pit, some ribs and vertebrae along with the right humerus and scapula were found in anatomical connection, confirming that the excavation team encountered the burial in primary context. However, the team documented some disturbance around the cranium, along the left part of the ribcage, and near the infant’s legs. The cranium was found “opened” with the left frontal flipped over the main cranial vault. Another cranial fragment was found ~ 5 cm north-east of the main vault. The slightly jumbled 3D position of most upper vertebrae and right ribs, as well as the lack or dispersal of most of the left ribs and lumbar vertebrae, further suggests that the core of the body was disturbed post interment. The disturbance found near the pelvis can confidently be associated with an animal burrow dug below the bones, where sediment and artifacts had started to erode north; excavation notes detail two elongated tunnels directly north of the cranium and the abdomen, with loose and dark sediment containing shells of land snails, which was very different from their surrounding matrix. In addition, a portion of the burial was also partially eroded by the surrounding gullies.

Small specks of ochre were found in the burial fill but the soil and the human remains were not stained red as seen in other burials (*e.g.*, Einwögerer *et al*., 2006; Henry-Gambier *et al*., 2019; Teschler-Nicola *et al*., 2020). Small chunks of ochre were found ~ 10 cm above the infant’s head; however, it is unclear if those were intentionally placed there in connection with the burial or if they are remains of older occupations included in the burial fill.

#### Grave Goods

**Fig. 4:**
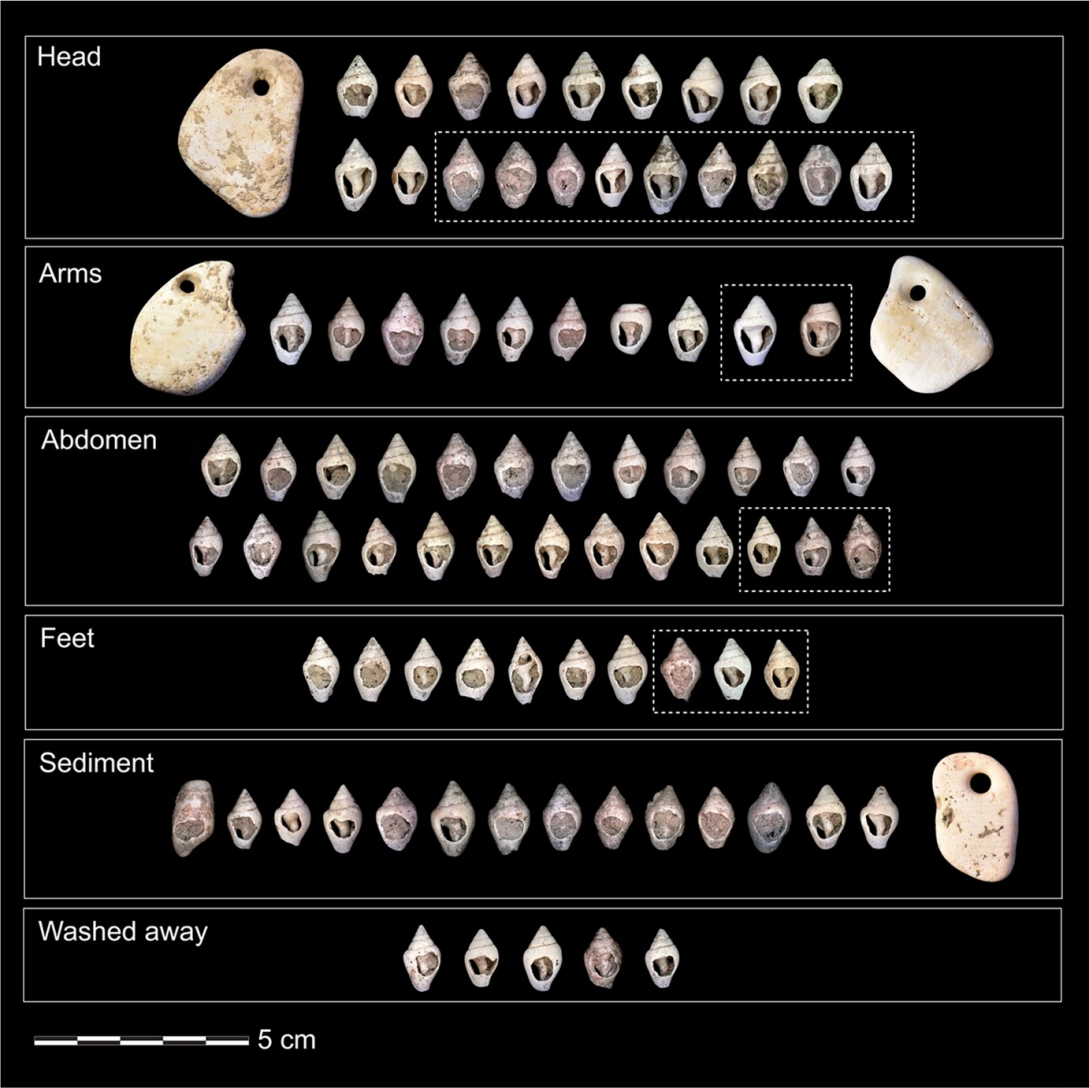
Personal ornaments found in and above the AVH-1 burial, grouped by where they were found in relation with preserved human bones (white boxes). The shells within dotted boxes were found within 3 cm of a human bone, but were not part of the recognizable arrangement formed by the other shells in that group. The shells found in the “Sediment” section were found > 3 cm from a human bone, whereas the shells in the “Washed away” section were found in a burrow located below and North of the burial pit

The human remains were accompanied by one lithic laminar flake, some ochre specks, and multiple beads made on perforated seashells of three distinct taxa. In addition, a nearby cache contained an eagle-owl talon, which has been interpreted as an offering (Hodgkins *et al*., 2021). The majority of the beads (*n* = 93) were made from *Columbella rustica*, one bead was made from a *Turitella* sp. shell, and four pendants were made on fragments of big bivalves that could not immediately be taxonomically identified (Fig. 4). Hodgkins and colleagues reported that AVH-1 was adorned with at least 66 *Columbella rustica*, which was based on CGM’s visual assessment of each bead’s position within the burial (2021). To get a more precise count for the present research, we computed the exact Euclidean distance between all beads and all mapped human remains. Using this method, we can confidently say that 71 *C. rustica* as well as three of the four pendants were found within 3 cm of a preserved human bone (see SI 3). However, some of the perforated *C. rustica* (*n* = 22)*,* the perforated *Turitella* sp., and one of the bivalve shell pendants were found in the burial fill, above the fill, or washed away in squares north of the burial.

**Fig. 5:**
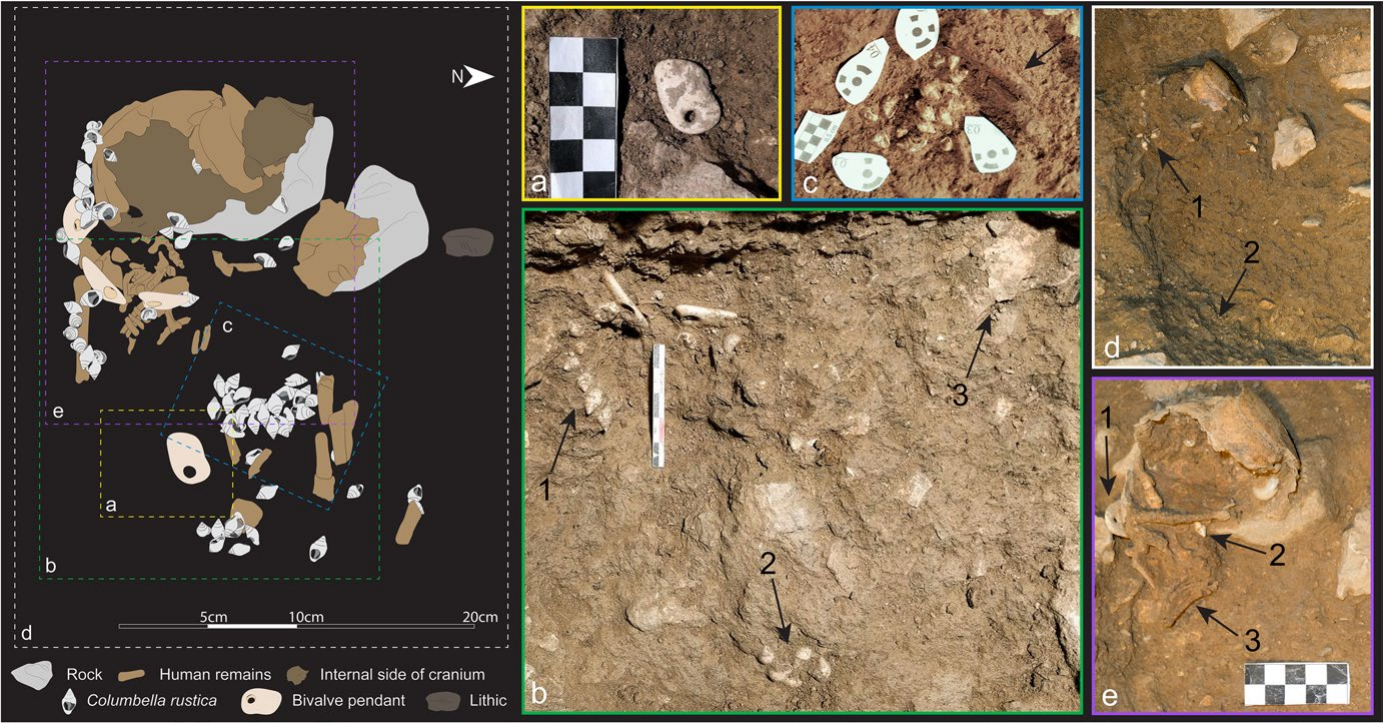
Field photos of the ornaments in the order they were excavated. **A** The first pendant found in 2017; **B** this 2017 photo shows (1) the location of the columbellae and pendants aligned around the right humerus and extending to the torso, (2) the cluster of shells found where the feet would have been, and (3) the displaced piece of cranium that was uncovered first. **C** As part of our team excavated down in 2018, they unearthed a cluster of shells next to a few pieces of long bone (arrow). **D** This shows (1) the line of beads found abutting the cranium and (2) the rough outline of the burial pit determined from sediment change. **E** The last part of the burial excavated was the deeper one, which includes (1) one of the pendants directly abutting the cranium, (2) perforated columbellae located directly underneath the cranium, and (3) multiple ribs

Most of the perforated shells found in direct association with the human remains can be separated into four clusters: one line to the right of the cranium (Fig. 5d1), one line that curls around the right humerus and likely continues onto the torso (Fig. 5b1), two lines located where the abdomen would have been (Fig. 5c), and a cluster of shells that were no longer aligned to form a distinguishable assemblage when excavated and were found above a human bone fragment that could not be assigned to a specific body part due to its poor state of preservation (Fig. 5b2). Given its 3D placement in relation to the long bones, we cannot exclude the possibility that it may be part of the right pelvic bone. However, to distinguish this last cluster from the one located near the abdomen, we label it “feet” as we hypothesize that the folded legs of the infant would have positioned the feet near her pelvis.

In the next section, we summarize the methods we used for our analysis of the ornaments found in the burial and our archaeothanatological study of the burial as a whole. We use the latter analysis to explore what the beads’ characteristics (3D position, use-wear, and ochre) tell us about the history of the burial, as well as the ways in which the ornaments may have related to the buried infant and her parents, in life and in death.

## Materials and Methods

### Microscopic Observations

All perforated shells found in association with the burial were inspected using a DinoLite Digital Microscope (~ × 20– × 150 magnification) in our field laboratory in Erli, Liguria. In addition, a selection of 60 *Columbella rustica*, the single *Turritella* sp., and the four bivalve pendants were subjected to more in-depth examinations at the DANTE—Diet and ANcient TEchnology laboratory (La Sapienza University of Rome)—using a Zeiss Axio Zoom V16 binocular stereo microscope with progressive magnifications ranging between × 10 and × 112, and equipped with a Zeiss Axiocam 305/506 color camera. These observations served to identify functional modifications (rounding, faceting, changes of color, striations, *etc.*) and residues on the analyzed artifacts. The extent and placement of those modifications and traces were drawn on an outline of each shell/pendant using Adobe Illustrator.

### GIS Analyses

To identify potential preferential placement of use-wear and residue traces on the shells, we adapted Marean and colleagues’ (2001) zooarchaeological approach of counting MNE to our study. With this method, the position of a bone fragment is drawn as a polygon (with a value of 1) onto a 2D vector template in ArcGIS. This is repeated with all fragments of the same bone type coming from one assemblage. The analyst can then sum the overlap of all fragments to calculate MNE for that specific bone type (*e.g.*, femur, scapula, *etc.*). For our research, we adapted this method by drawing the extent of the use-wear and residues on each *C. rustica* instead of the extent of the shell itself. The drawings done in Adobe Illustrator were exported as vector polygons with a value of 1 into ArcMap 10.6, where they were georeferenced onto a 2D template shell outline. We were then able to sum the values of all polygons grouped by placement on the infant’s body to get a summary of where use-wear and residues were most often found.

### Micro-CT for Morphology and Use Wear

All excavated shells were micro-CT scanned at the Institute of Clinical Physiology (CNR-IFC), in Pisa. The shells were grouped in batches of 6–8 using a polystyrene foam holder allowing proper spacing and individual labeling of each shell; each batch was then scanned with a IRIS-CT tomograph (Inviscan SaS, Strasbourg, France) with the following settings: 80 kV, 1 mA, 2000 projections over 360°, 60 ms of exposure time per projection. Volumetric images were reconstructed using cone beam–filtered backprojection (FBP), with an isotropic voxel size of 58.8 μm. After reconstruction, the 3D image of each shell was then cropped from the global (batch) image, assigned non-ambiguously to the specific shell label with the aid of radiolucent markers placed on the holder and then exported to a stack of TIFF slices. The resulting stacked TIFF were processed in Amira to produce 3D polygon models as.ply files. The scans can be found on tDAR (https://core.tdar.org/project/468844/arma-veirana-excavation).

**Fig. 6:**
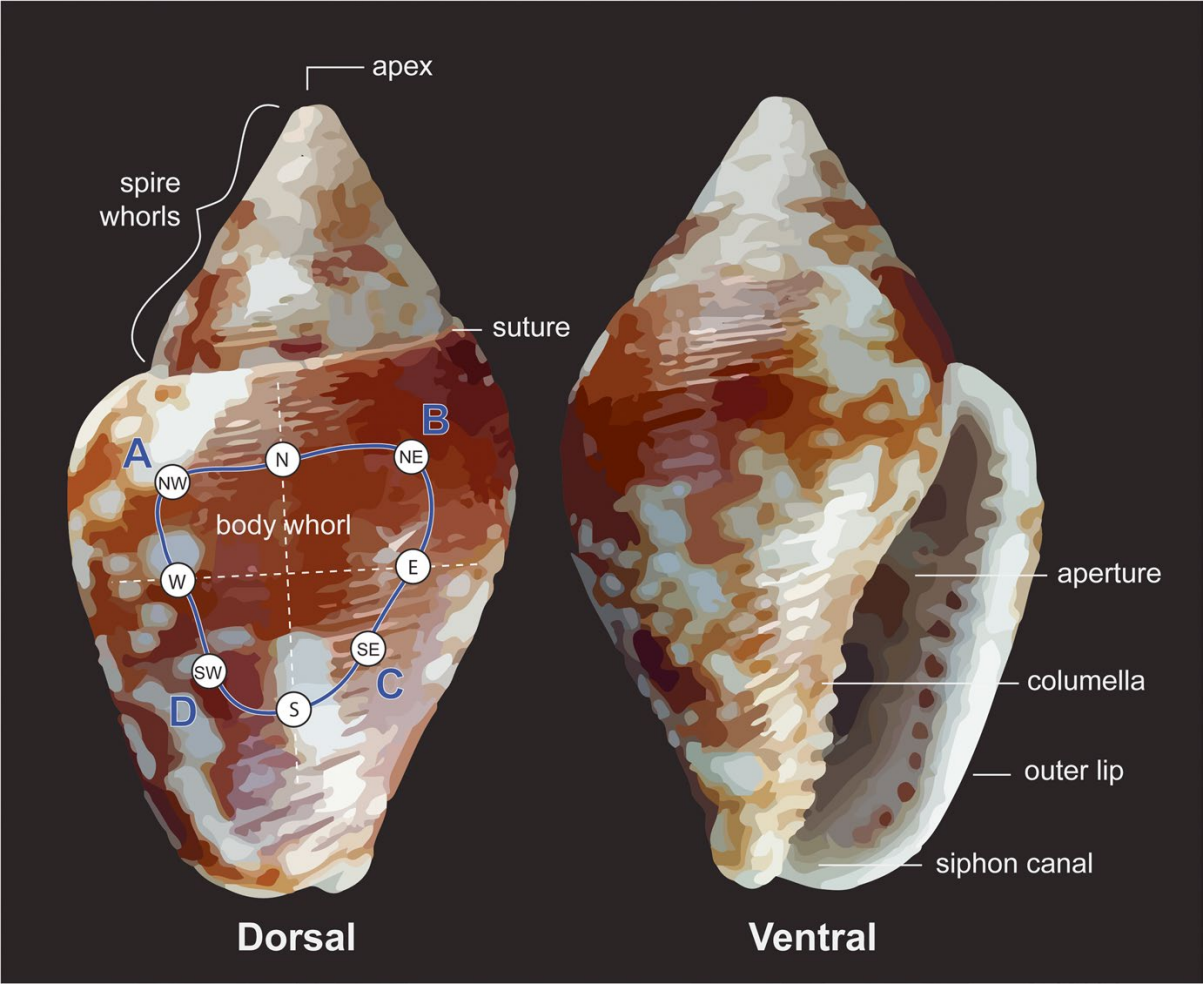
Anatomical parts of *Columbella rustica* shells. The blue outline on the dorsal side represents the general position of most perforations. The letters in blue (**A**–**D** on dorsal) show the four quadrants where surface roughness score was quantified in CloudCompare, whereas the encircled cardinal directions show where the edge angle was quantified in Meshlab

We used the free Meshlab software to analyze the models in different ways. We first took morphometric measurement of all *C. rustica*, which we compared to measurements of modern specimen aggregated from a literature review (Benghiat *et al*., 2009; Cartonnet, 1991; Perlès, 2018). We then computed a relative rounding value of each ornament’s perforation using the Virtual Goniometer plug-in (Yezzi-Woodley *et al*., 2021). This process relied on the assumption that shells with longer use would have more rounded perforation edges than shells that had recently been perforated. For this process, we first measured the angle of the perforation edge at eight positions around the perforation (N, NE, E, SE, S, SW, W, and NW) (see Fig. 6). For each cardinal position, we then normalized the angle of all shells to a range of 0–1. Finally, for each shell, we computed the sum of the normalized measures, which was used as its use-wear score.

In addition to measuring the angles, we computed quantitative data obtained through 3D 360° analysis of the perforation holes. Following established protocols (see Cristiani *et al*., 2021; Zupancich & Cristiani, 2020), we examined the surface roughness around the shells’ perforations, with the assumption that differences in surface roughness values reflect differences in degree of wear development. In theory, areas of the perforation characterized by highly developed use traces should exhibit a more homogeneous surface, resulting in low values of roughness. Conversely, the areas with less-developed traces should show a more heterogeneous surface, resulting in higher roughness values.

Roughness values were computed on the micro-CT scans of the *Columbella rustica* shells bearing wear traces (*n* = 76). In some instances, the 3D model of the columbellae also included some extraneous elements, such as sediment particles entrapped in the perforation. Because those elements would have affected the recording of surface roughness values, the *Select Faces* tool in Meshlab v.2022.02 (Cignoni *et al*., 2008) was used to manually remove all such elements. After this cleaning step, the scans were imported in CloudCompare (v.2), where they were segmented to isolate the region surrounding the perforation from the rest of the shell body. The region around the perforation was then divided into four quadrants (upper left, upper right, lower left, and lower right) determined by its longer and shorter axes (see Fig. 6). Roughness values were then computed using a neighbor radius of 1 mm. To perform statistics and reduce the noise in the data, we sampled the raw data to keep only 100 points from each quadrant for each shell. We also computed the mean of these sampled points for each shell to obtain a second use-wear score.

We computed Wilcoxon rank sum tests with Bonferroni adjusted *p*-values in R (version 4.0.4, R Core Team, 2019) to compare the two computed use-wear score of shells found in association with different parts of the body (head, arm and torso, abdomen, and feet). We also calculated the Pearson’s correlation between the shell’s use-wear scores and their sizes (length, thickness, and perforation size). The R scripts used for these analyses can be found in SI.3, whereas the raw data is available on https://github.com/cgravelm/Arma_Veirana_ornaments.

### Taxonomy

To identify the taxon of the shellfish used to make the pendants, we first used morphometrics and microscopic analysis to select a set of features that were found on all specimens (see “Results” section below). We then used a tumbling experiment on specimens of modern shells from potential taxa.

As the archaeological specimens were fragments of valves, we first used a hammerstone to break the modern specimens into pieces of roughly similar size to the archaeological pieces. The resulting pieces were photographed, measured, and then placed in a Lorotone 3A rock tumbler with 1.5 lb of ceramic media and 2 tbs of coarse grit. The tumbler was run continuously for 4 days. The Lorotone 3A has a 10 cm diameter barrel and runs at 60 rotations per minute. Following the equation used by Gorzelak *et al*. (2013), 4 days at this tumbling speed has the same eroding effect as surf zone movement over ~ 57.6 km. After 4 days, the tumbled fragments were washed, photographed, measured, and observed using a DinoLite Digital Microscope. The fragments were then placed back into the tumbler for an additional 4 days with the same ceramic filler tiles and 2 tbs of medium grit. After this step, fragments were again washed, photographed, measured, and observed under the microscope. The tumbling exposed the shell pieces’ internal structures similar to those visible in the archaeological specimens. We compared microscope observations of the tumbled fragments to the observations of the archaeological sample and noted similarities in finish, shape, and size to identify the taxon from which the archaeological ornaments were made.

### Preliminary Perforation Experiment

Two of us (CGM and JRS) performed a quick, unregularized experiment to explore ways in which the pendants might have been perforated. For this experiment, we used tumbled fragments of *Glycymeris* bivalves and attempted to perforate them using lithic drills. The results were observed macroscopically and compared to the patterns seen on the archaeological pendants.

### Burial Context

To reconstruct the funerary practices that took place at Arma Veirana and understand the relationship between the buried infant and her body ornaments, we used analytical techniques derived from the “Anthropologie de terrain,” otherwise known as archaeothanatology, a discipline that combines field documentation with knowledge of how dead bodies decompose (Duday *et al*., 1990; Nilsson Stutz, 2006). This analysis combined data from the 3D photogrammetry model, GIS data, and field photos to document the relative elevation of all components of the burial, which allowed us to reconstruct in detail the superposition of each element and better document how they relate to one another (see S1 video) and analyze the ornaments’ use-wear and residue placements within each arrangement. One bivalve pendant (PF #3484, see Fig. 5a) was recovered 6 cm directly above the remains before the excavation team found the first cranial piece; therefore, it was not imaged using the photogrammetry methods used to document the rest of the burial. To document its spatial relationship with the burial, we relied mainly on GIS data and georectified field photos.

## Results

### Bivalve Pendants

**Fig. 7:**
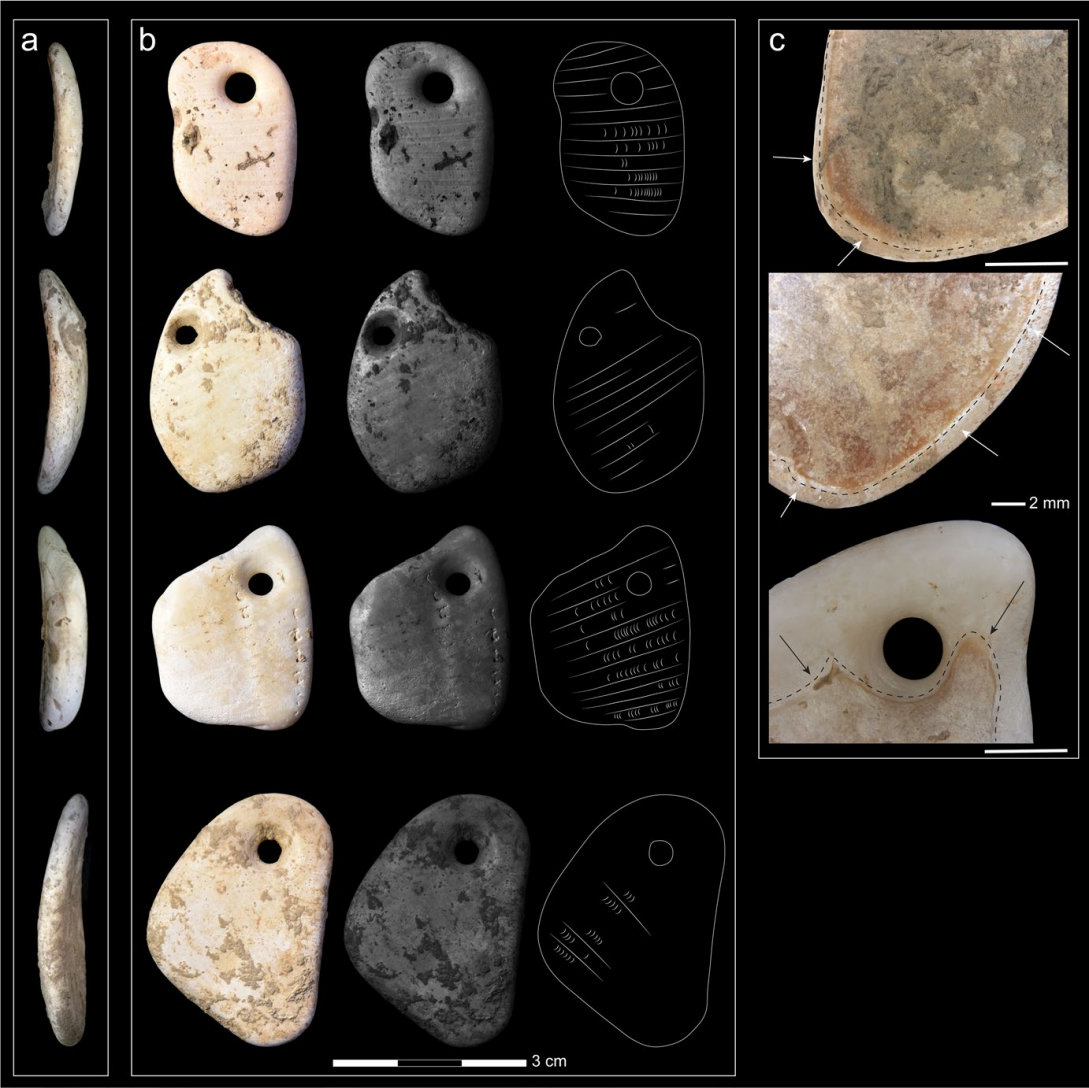
Characteristics of the pendants. **A** Side view. All pendants have a smooth and regular surface with a slight convex curvature and are quite thick. **B** This portion shows the dorsal side of all pendants (left), a black-and-white version of that image with enhanced contrast to show the ribs (middle), and a tracing of the ribs done using the BW image (right). **C** Three out of four pendants have a natural red layer on their ventral side. Their extent is shown here with the dotted lines (the scale bars represent 2 mm)

The four pendants have a roughly similar oval to quadrangular shape (see Fig. 7) and measure 33 × 23 × 4 mm on average (Table 1). They have heavily rounded edges and a uniform polish, with natural red portions on the ventral side. As they retained a certain transparency, we can safely assume that those specimens were fresh marine shells rather than fossils (see Dimitrijević & Tripković, 2006).

**Table 1:** Morphometric measurements of the four pendants

Specimen number	Length (mm)	Width (mm)	Thickness (mm)
3484	31.4	19.8	3.9
3854	34.2	23.7	3.8
3858	29.8	22.8	4.6
9228	37.8	26.3	4.1

Macro- and microscopic analyses showed that all pendants had the following characteristics: 1. the dorsal surfaces of the fragments were smooth, regular, and with a slight convex curvature (Fig. 7a); 2. the ribs visible on the fragments were regularly spaced and converging toward one of the sides (Fig. 7b); 3. the ventral side of most fragments retained natural red features (Fig. 7c); and 4. all fragments were very thick (mean 4.1 mm) (Fig. 7a).

To identify their taxon, we looked for those characteristics in the tumbled specimens of different taxa. We tumbled fragments of *Glycymeris* sp.—including *Glycymeris glycymeris* and *Glycymeris pilosa*—and *Arctica islandica* because they are of the same size and shape as the pendants, fragments of *Haliotidae* (abalone) and *Ostrea* (common oysters) due to the presence of iridescent sheen on the pendants, and *Spondylus gaederopus* because of the red ventral coloring found on the pendants and because *Spondylus* was a widely used taxon traded during the Late Mesolithic and Neolithic (Dimitrijević & Tripković, 2006).

**Fig. 8:**
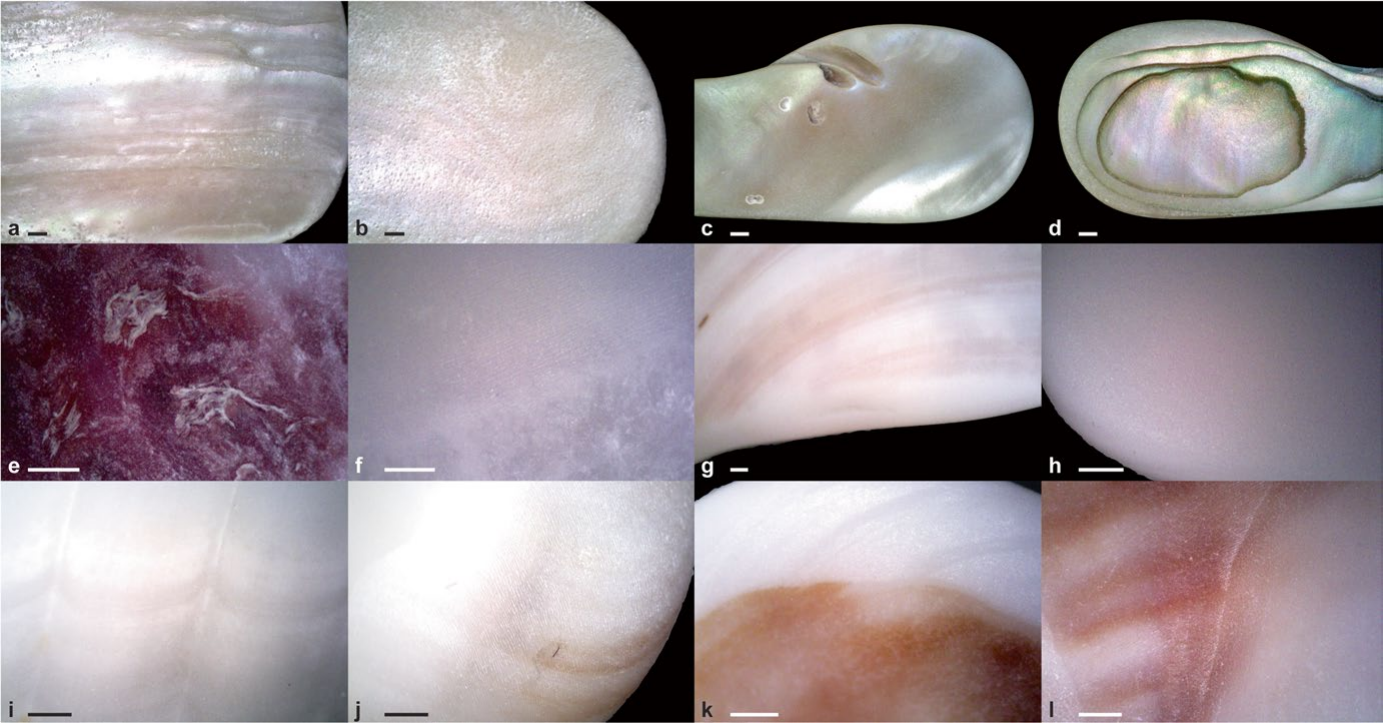
Tumbled bivalves observed after eight days in the tumbler. **A** and **B** *Ostrea*, **C** and **D** *Haliotidae*, **E** and **F** *Spondylus gaederopus*, **G** and **H** *Arctica islandica*, **I** and **J** *Glycymeris glycymeris*, and **K** and **L** *Glycymeris pilosa*. For each taxon, the images are of the dorsal and ventral sides, in that order. The scale bars represent 1 mm

The results of this experiment (Fig. 8) suggested that all pendants were made on fragments of *Glycymeris* sp*.* as it was the only taxon that had all the characteristics enumerated above (Table 2); *Glycymeris* pieces became smooth fragments with slightly convex regular surfaces, visible converging ribs, and red ventral pattern similar to what was found on the archaeological pendants. As a contrast, tumbled fragments of *Spondylus gaederopus* shells retained the bumpiness of their dorsal sides, their ribs were sinuous rather than straight, and they had a red hue on their dorsal side, but their ventral side was pearly white. Moreover, tumbling the *Glycymeris* pieces increased the visibility of the sheen formed by the inner complex crossed-lamellar layer, which corresponds to the sheen observed on the AVH-1 pendants. A few fragments of *Spondylus gaederopus* displayed that similar sheen, but not as consistently as *Glycymeris* fragments. Therefore, our experiment strongly suggests that the pendants found in association with the burial were made from pieces of *Glycymeris* valve.

**Table 2:** Presence or absence of pendants’ diagnostic characteristics in tumbled bivalve fragments

Tumbled taxon	Smooth, regular, and with a slight convex curvature on dorsal side	Ribs visible, regularly spaced, and converging towards one of the sides	Natural red coloring on ventral side	Thickness (average after tumble)
*Arctica islandica*	Yes	Yes	No	2.7 mm
*Glycymeris glycymeris*	Yes	Yes	Yes	3.6 mm
*Glycymeris pilosa*	Yes	Yes	Yes	3.6 mm
Haliotidae	No	No	No	2.7 mm
Ostrea	Yes	No	No	2.2 mm
*Spondylus gaederopus*	No	No	No	5.3 mm

#### Use-Wear and Perforation

**Fig. 9:**
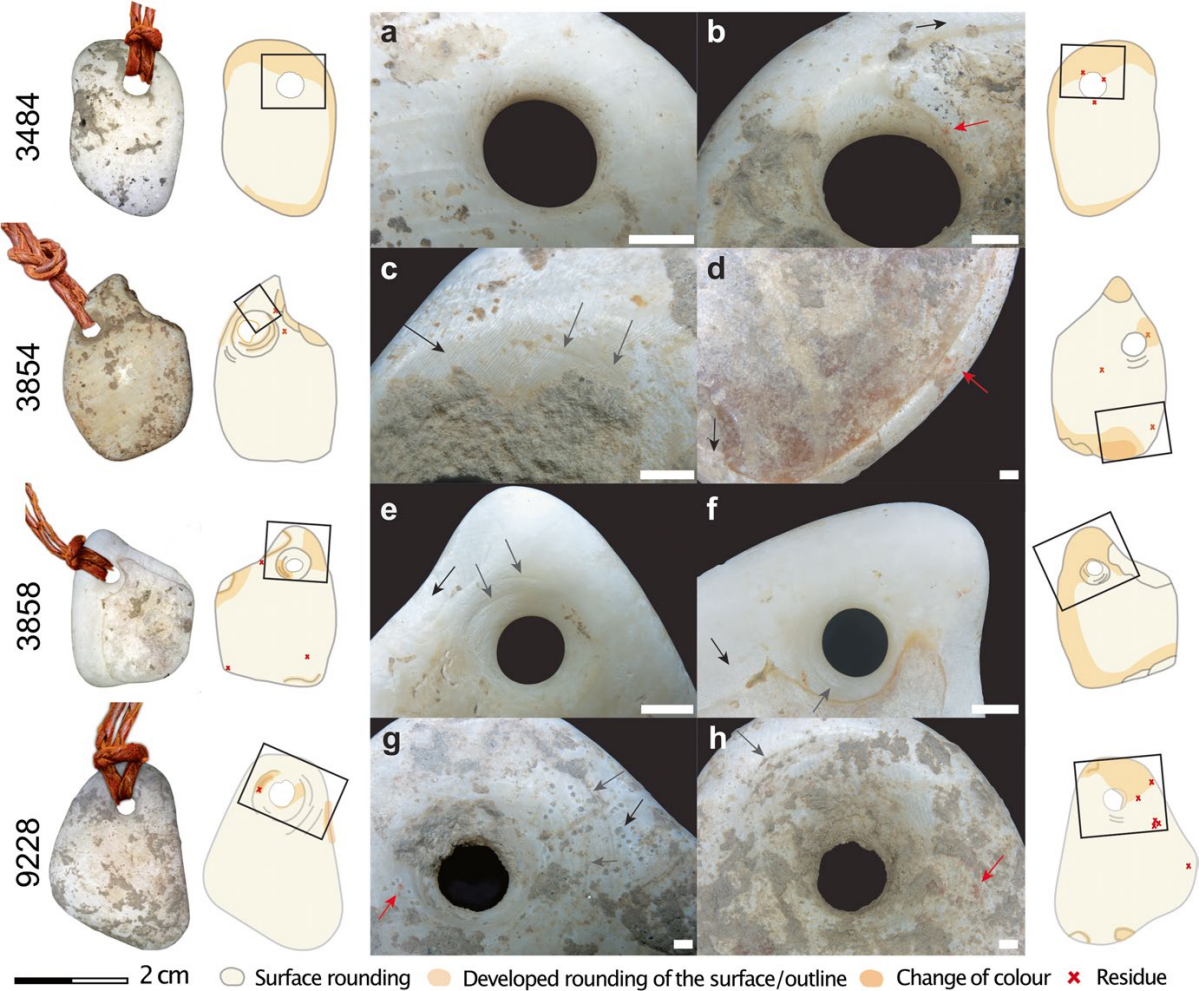
Multiple aspects of the AVH-1 pendants. On the left is an interpretation of how they might have been suspended. The figure includes drawings of the distribution of use-wear and residues (on the dorsal side on the left and ventral side on the right) accompanied by microscopic images of key parts of each pendant (dorsal on the left and ventral on the right). On microscope images, the gray arrows point to perforation striations, the black arrows to the natural sheen, and the red arrow points to ochre residues. In those photos, the white scale bar measures 1 mm

The observations done with the Zeiss AXIO ZOOM V16 showed the presence of developed rounding on the ventral surfaces (Fig. 9a, c, e, and f) and edges of all pendants (Fig. 9d–f, and h) as well as on the perforation walls (Fig. 9a, b, and e–g), indicating that the ornaments were worn with their ventral side rubbing against a pelt or skin for a considerable amount of time before being buried with the infant. In addition, the presence of different levels of roundedness on the fragments’ edges of two pendants (3854 and 3858) suggests that these two fragments may have been collected on the beach already rounded by wave action, knapped to a desired form, and then worn for a substantial period of time. The use-wear located in and around the perforations falls at the natural location one would expect given the weight and shape of the pendants. Ochre is found episodically on the pendants, with a somewhat preferential placement around the perforation.

The presence of circular striations around the perforations demonstrates that all four pendants were perforated using bifacial drilling with a lithic implement (Fig. 9). In three of the four pendants, the dorsal cone is larger than the ventral cone, which indicates that the former was drilled longer than the latter side. The preliminary perforating experiment done on tumbled *Glycymeris* fragments suggests that this differential time may simply be the result of working constraints caused by the convexity of the shell. Drilling the dorsal side with the edge down offers a stable surface that can be pierced through, with the perforation being subsequently finished by drilling and regularizing the opening from the ventral side. Beginning the perforation on the ventral side was much more involved due to the convex face wobbling on the surface during work.

### Perforated Columbella rustica

#### Morphometrics

*Columbella rustica* (Linnaeus 1578) are omnivorous gastropods of the Buccinacea family. They are characterized by their ovoid and pointy shape, five whorls including the body that typically measure ~ 2/3 of the full length, and an elongated, wavy opening with teeth (Álvarez-Fernández, 2006; Taborin, 1993). Modern specimens collected on the Adriatic coast and on the coast of the Gulf of Lion reportedly measure on average ~ 13.5–14 × 8–9 mm (Benghiat *et al*., 2009; Cartonnet, 1991), whereas specimens collected in Greece recently were a bit smaller (11.4–11.7 mm in Perlès, 2018).

The *C. rustica* found at Arma Veirana have an average length and width of 14 mm and 8.4 mm, respectively (Table 3), which falls either within the modern size average mentioned above or are slightly bigger, depending on which control sample we use. The perforations of all shells are relatively large, covering on average 38% of the body whorl’s dorsal face.

**Table 3:** Morphometric summary of *Columbella rustica*. The perforation ratio refers to the percentage of the dorsal body whorl covered by the perforation

	Min	Mean	Max
Length	11.0 mm	14.0 mm	17.1 mm
Width	7.2 mm	8.4 mm	9.9 mm
Perforation ratio	25%	38%	73%

**Fig. 10:**
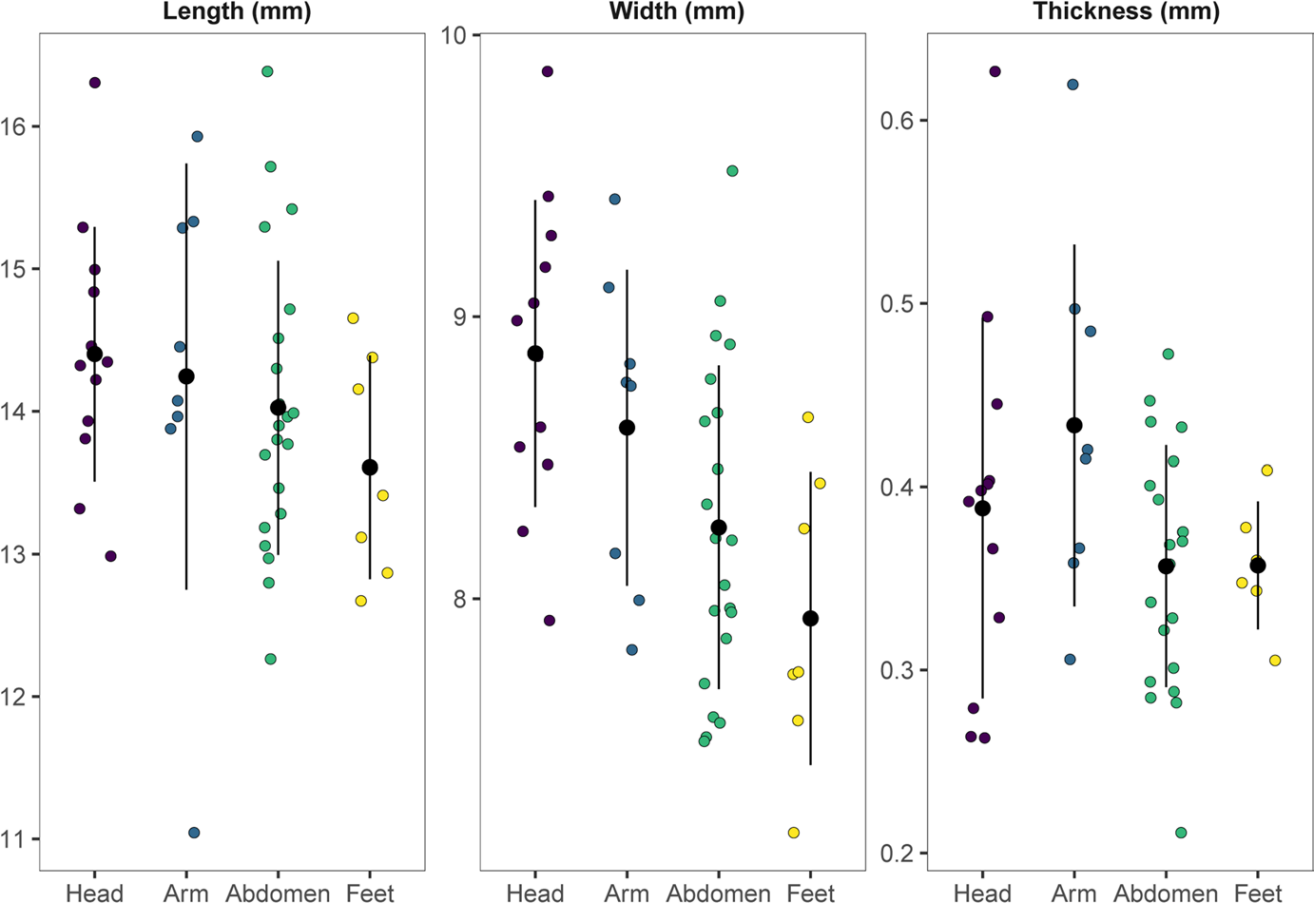
Length, width, and thickness of the *C. rustica* beads found in recognizeable arrangements in direct contact with the human remains. Sample sizes = head (12), arm (10), abdomen (22), and feet (7). The black dots and lines represent the mean and one standard deviation around the mean

When aggregating the beads by their position in the burial—focusing on the shells that were found in recognizeable arrangements—and comparing their means using Wilcoxon rank sum tests, none of the grouped average dimensions are statistically different (Fig. 10).

**Fig. 11:**
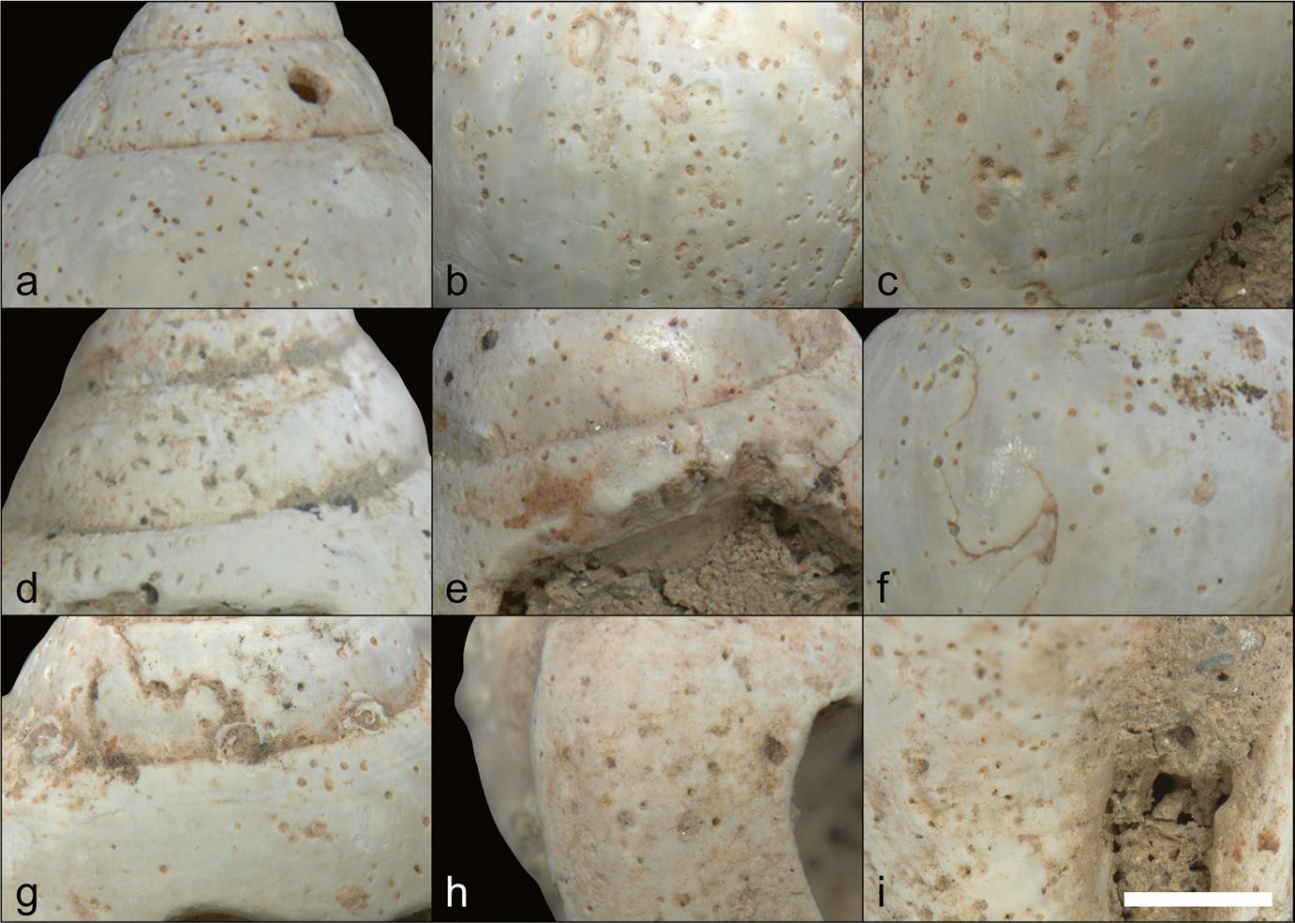
Multiple examples of sponge marks indicating that the shells were collected on the beach. All photos are on the same scale and the white bar represents 2 mm

**Fig. 12:**
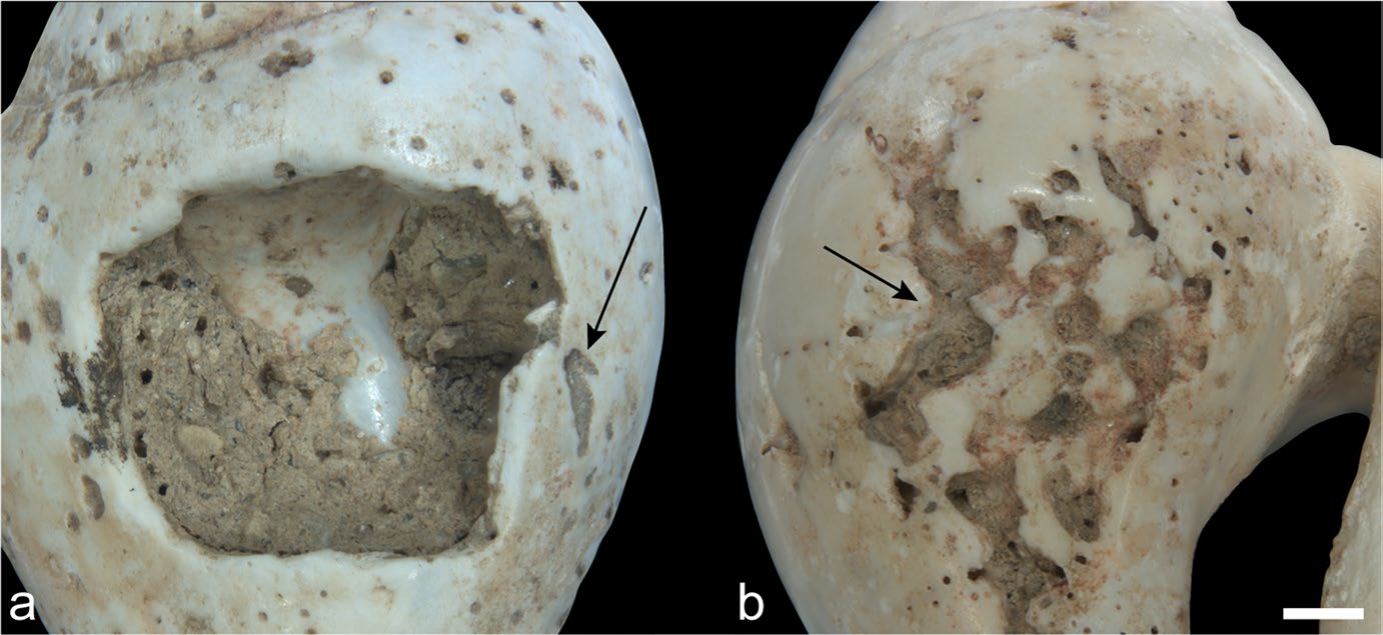
Example of root damage found on a few shells. Both images are on the same scale and the white bar measures 1 mm

Visual observations of all shells show that 77 *C. rustica* have sponge marks and other forms of pitting that indicate that they were collected from modern thanatocenosis on the beach (Fig. 11). Moreover, while a few specimens were very well preserved, most *C. rustica* were poorly preserved, with some root damage (*n* = 9 shells) (Fig. 12) and decalcification.

#### Perforation Method

Microscopic analysis shows that most *C. rustica* found at Arma Veirana bore large quadrangular perforations with straight edges. Crushing on the upper area of the perforation (on the line going from NW to NE shown in Fig. 6) was observed in a few specimens, which suggests that the perforations may have been produced on the shells’ exterior by direct percussion using a hammerstone or a flint core.

#### Use-Wear

**Fig. 13:**
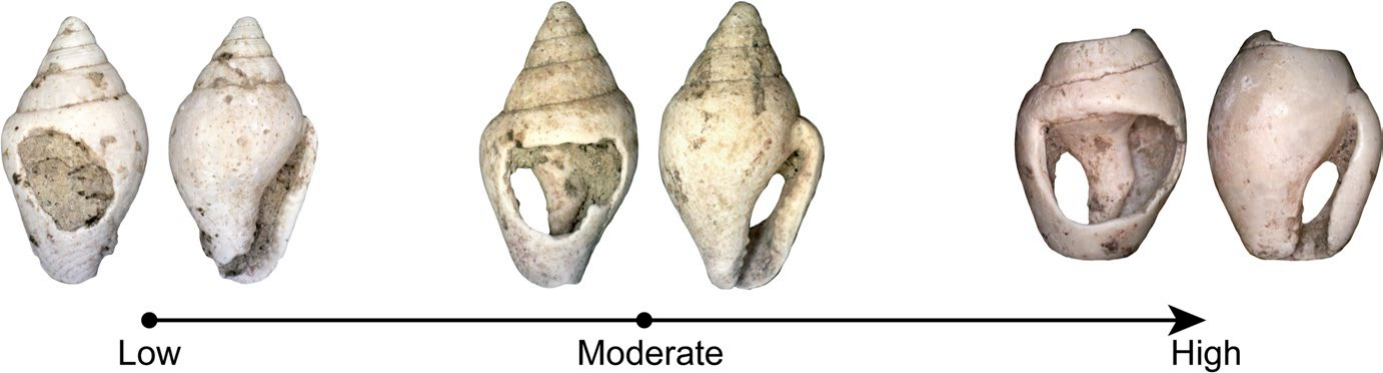
Range of use-wear seen in the shells. The mean surface roughness values of the shells from left to right are 0.019, 0.015, and 0.013

**Fig. 14:**
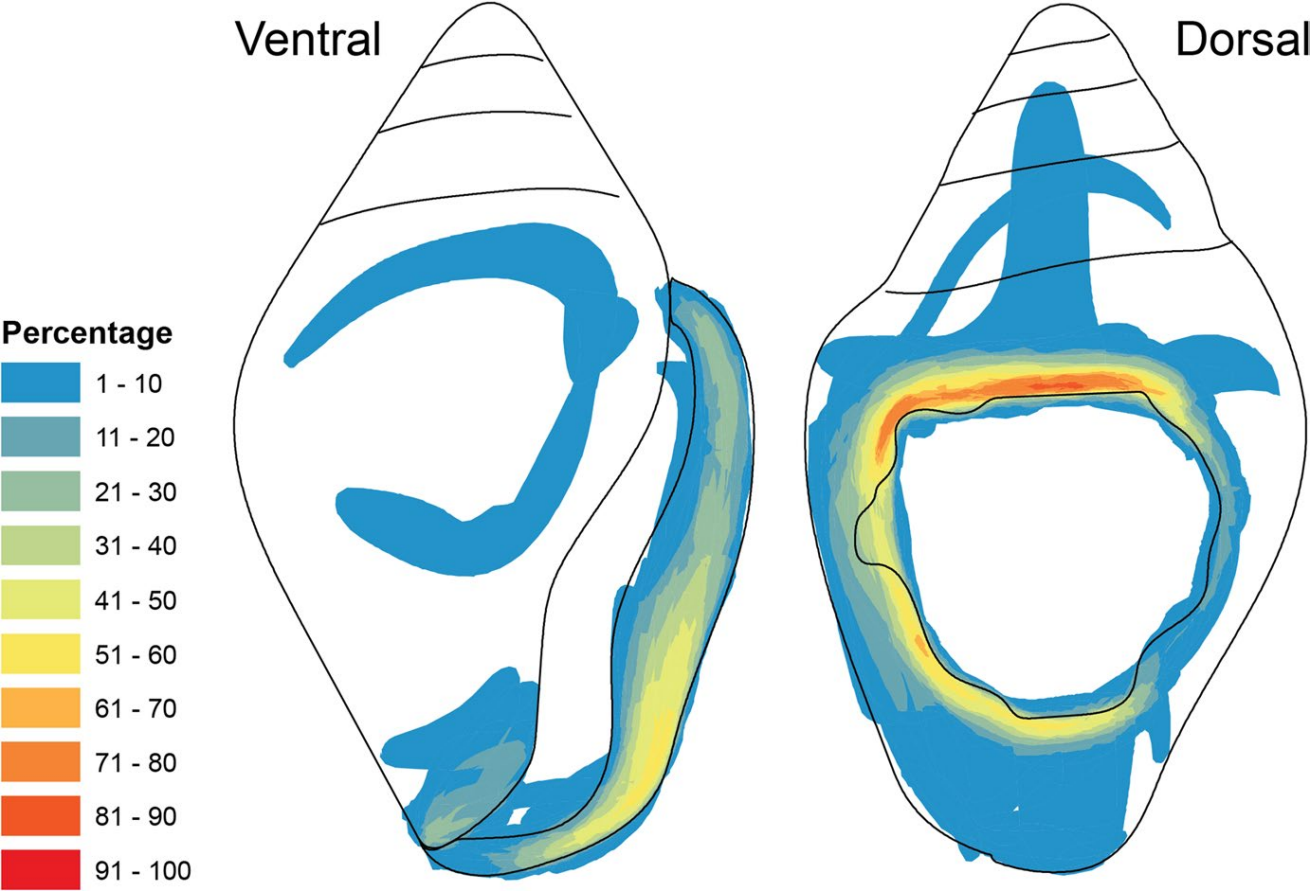
Percentage of shells with use-wear on each location (shells in arrangements only)

Microscopic analysis shows that the perforations of most shells were very worn (see Figs. 4, 11e, and 13). On the shells found in arrangements, use-wear is most frequent on the left and top left portions of perforations as well as on the lower half of the natural aperture (~ 70–100% and ~ 60% of shells, respectively, see Fig. 14). In some instances, the use-wear develops invasively on the lateral side, from the top left of the perforation to the bottom of the natural aperture, whereas in others, the use-wear is found only on the left parts of the perforation without noticeable traces on the natural aperture. In addition, natural growth lines are often preserved on the outer lip. These suggest that the string used to attach the shells was not tied around their outer lip, but rather went directly through the perforated shells sometimes obliquely and sometimes relatively straight through the openings.

**Fig. 15:**
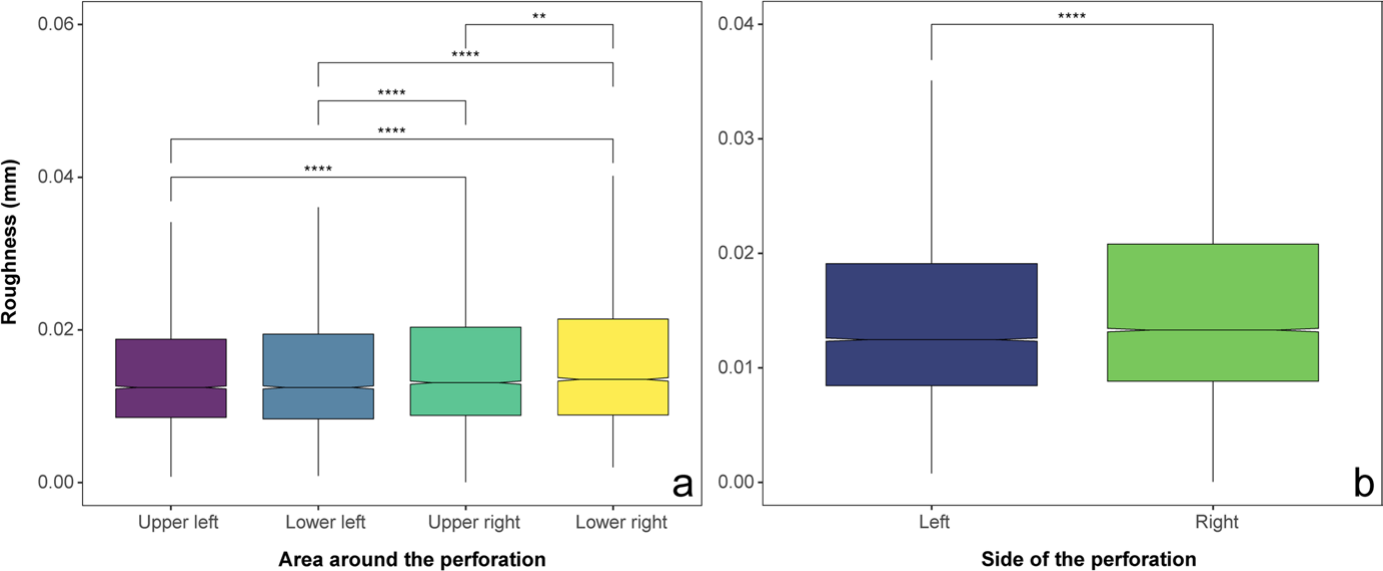
Average of the surface roughness measured on the 3D scans using CloudCompare. Measures aggregated by quadrants (**A**) or by the perforation side (**B**). Statistical different averages are indicated by the significance symbols (**: *p*-value <  = 0.01, ****: *p*-value <  = 0.0001)

The surface roughness score computed on the micro-CT confirms the pattern mentioned here. Surface roughness values are significantly lower on the left side than on the right side of the perforation (Kruskal–Wallis chi squared = 81.86, df = 1, *p*-value < 0.001), with the upper left quadrant having significantly lower roughness than both right quadrants (see Fig. 15). Moreover, the roundedness and average roughness use-wear scores show that use-wear intensity differs significantly by placement in the burial, with shells used for the head arrangements showing the highest levels of use-wear (Table 4). However, shells with different levels of use-wear are also mixed within singular bead arrangements (see SI4).

**Table 4:** *P*-values of the pairwise Wilcoxon tests comparing the average roughness and the roundedness of shells’ perforation grouped by their placement on the body. Statistically significant *p*-values are shown in bold; those suggest a significant difference in the use-wear values between shells placed on those two body regions

	Mean surface roughness			Summed angles (controlled for thickness)		
	Head	Arm	Abdomen	Head	Arm	Abdomen
Arm	**0.033**			0.128		
Abdomen	0.146	1.000		0.060	1.000	
Feet	**0.008**	1.000	0.472	**0.004**	1.000	0.115

Average roughness and summed angles do not correlate strongly with shell size (Pearson’s *R* =  − 0.19, *p*-value = 0.09 and *R* = 0.20, *p*-value = 0.06, respectively) but they do correlate significantly with the size of the perforation area (Pearson’s *R* =  − 0.32, *p*-value = 0.005 and *R* = 0.49, *p*-value < 0.001, respectively). Summed angles also correlate significantly with thickness (Pearson’s *R* =  − 0.36, *p*-value = 0.001)

#### Facets and Fractures

**Fig. 16:**
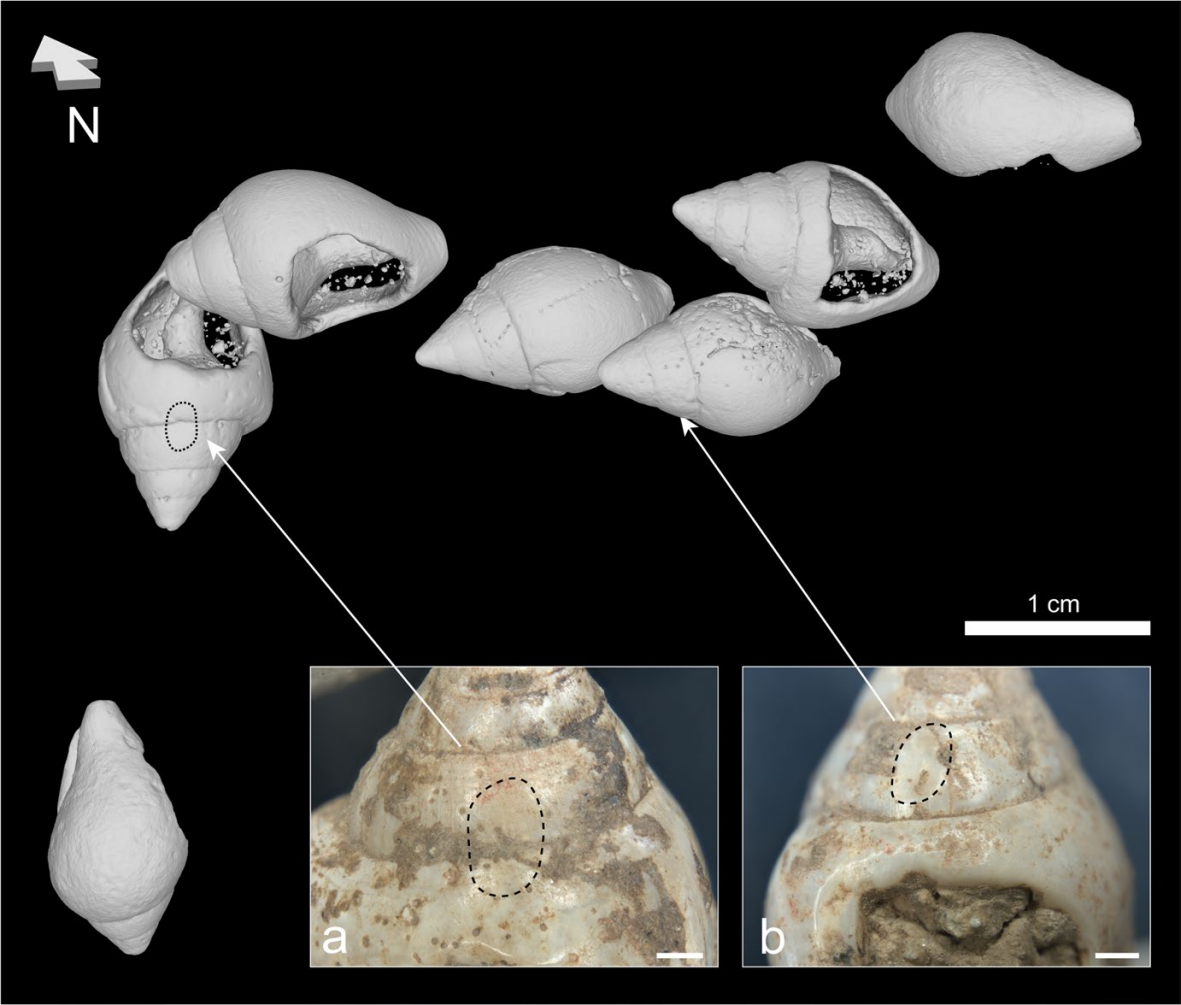
3D position of beads found aligned near the head. This image uses the micro-CT scans positioned based on the 3D photogrammetry model. The dashed lines and arrows show the location of the two facets mentioned in the text, with their microscopic images (**A** artifact #6262; **B** artifact #6261). The scales for the two microscope images measure 1 mm

**Fig. 17:**
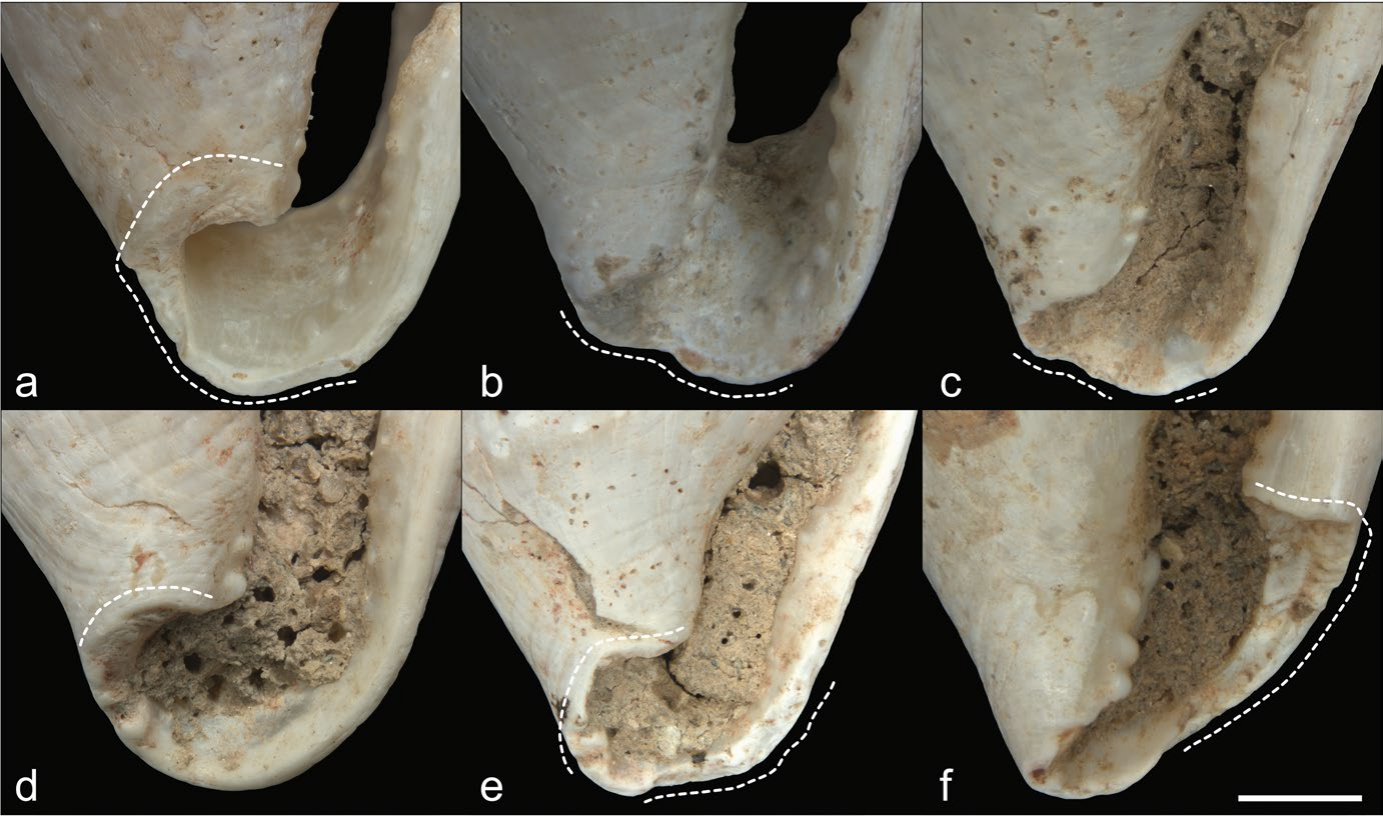
Multiple example of fractures at the bottom of the shell. All images are on the same scale and the white bar measures 2 mm

Two shells found near the head had wear facets on their spires (Fig. 16). In addition, fractures are somewhat common in the perforated shell assemblage (Fig. 17). Most of the shells found around the right humerus had a fracture at the bottom of their siphon canal, most of which were rounded. Fractures are also frequent on the shells found near the head, the abdomen, and the feet. In all arrangements, most fractures are found on the bottom of the shells (*n* = 35) (although two were found on the outer lip, 1 on the perforation, and 1 on the main whorl) and are almost always rounded.

#### Residues

**Fig. 18:**
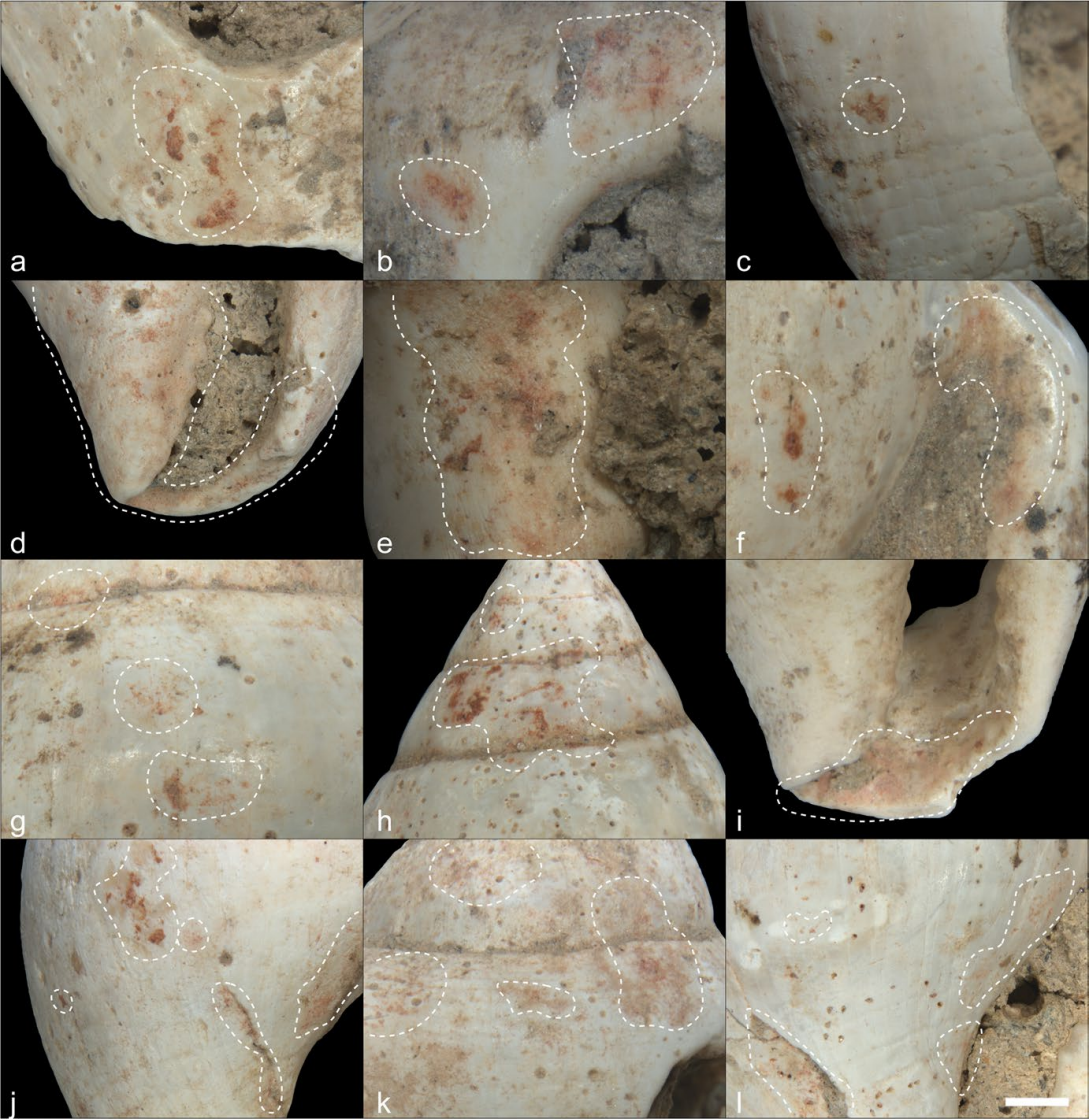
Multiple examples of ochre residues documented on the shells. All images are on the same scale and the white bar measures 1 mm

Specks of ochre residues were documented on 68 *C. rustica*, especially in the sutures of their spires, on the internal side of the columella visible through the perforations, and on the left bottom side of the perforations (Fig. 18).

The extent and concentration of ochre vary slightly per arrangement (see SI 5). Ochre residues are found on most shells located near the head and the “feet,” but in light quantities. Residues are most present at the top of the aperture on the ventral side, as well as on the dorsal body whorl and on the internal columella. The shells found around and near the left arm have specks of ochre on all sections of their surface, with slightly higher densities on the sides of the natural aperture. However, these shells have considerably fewer ochre traces than the shells found next to the head and have almost no residues on the columella. Ochre is prevalent everywhere on the abdomen shells, including on the internal section of the columella visible through the perforation. Multiple shells have ochre traces on the top and bottom of the aperture (*e.g.*, Fig. 18d, f, i, and l).

In the next section, we analyze the characteristics of the burial as a whole and we combine that information with the bead use-wear and residue information provided here to document how the ornaments were likely worn by the infant.

### Burial Context

As mentioned above, the infant was buried in a very small pit (~ 30 × 15 cm) constrained at the north and south by two stones that were too large to have been part of the burial fill (see Fig. 3). Radiocarbon dating of faunal remains and charcoal pieces found near the human remains combined with micromorphological analyses of the sediment strongly suggests that the pit was dug near the time of burial, and that the body was covered with the removed sediment (Hodgkins *et al*., 2021). Our archaeothanatology analysis of the 3D position of the human remains and the grave goods presented here supports this conclusion and provides more details about how the funeral may have taken place. To illustrate the descriptions provided below, we provide images from the 3D photogrammetry model, which show the distribution of artifacts and human remains better than field photos due to the poor preservation of some of the remains.

**Fig. 19:**
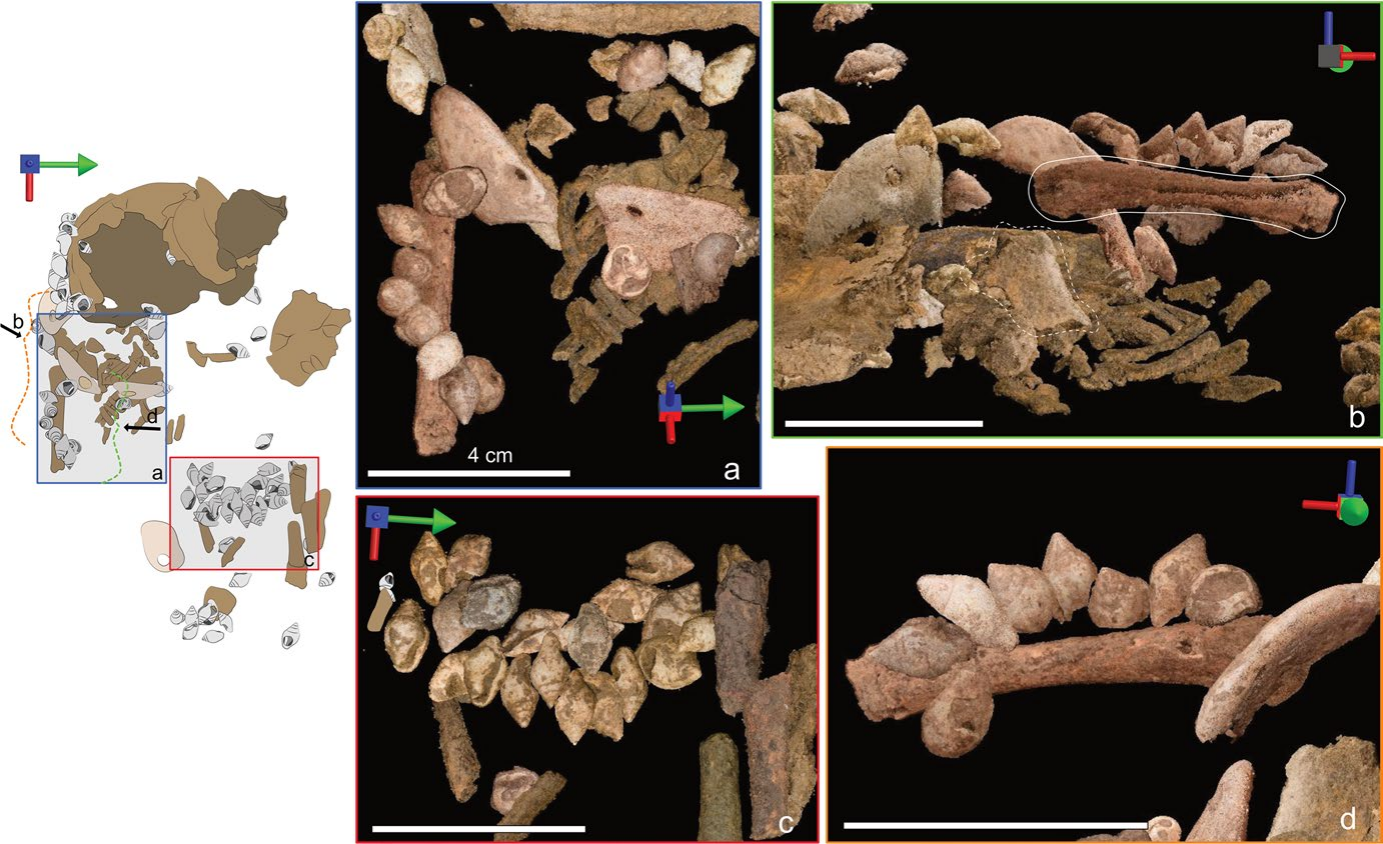
Parallel-projection orthographic views of artifacts and human remains from the photogrammetry model. The white bar measures 4 cm for all images, and the multidirectional arrow shows the viewpoint of the image, where the green arrow points to north, the red to east, and the blue points up. The drawing on the left can be used as a reference to identify the location of each image. Rectangles relate to views of the scan seen from above, whereas the dotted curly brackets show the viewing angle of the scan viewed from the side. **A** The relative position of the shells found in situ wrapping over the right humerus. **B** The relative position of the scapula (surrounded by the dotted contour) and the right humerus (full line). **C** Relative position of the perforated columellae found in the abdomen area. **D **Relative position of the perforated columbellae and pendants found near the arm and torso

Archaeothanatology shows that as the body decomposes, soft tissues leave empty spaces around bones, thereby destabilizing them (Duday *et al*., 1990). However, when a body is covered with sediment, some of the sediment will move into those empty spaces, thus preserving the initial position of the bones (Dedet *et al*., 1991). In this case, the 3D placement of the right humerus and scapula—found in anatomical position but higher than the ribs—follows this pattern. Moreover, the shells found around the right humerus as well as the abdomen seem to have preserved their position relative to one another, which would not have occurred in an empty pit (Fig. 19).

In archaeothanatology, the position of certain bone connections can be used to evaluate if a body was wrapped or interred in a confined space. Unfortunately, due to the poor preservation of the bones, we were not able to document the 3D position of some of these specific connections or of the clavicle, which is often a clear indicator of wrapping. The higher elevation of the right humerus in relation with the ribs (Fig. 19b) suggests the use of wrapping, because wrapping has been shown to project the shoulders forward and upward (Nilsson Stutz, 2006). However, this elevation could also be due to the proximity of the pit sides to the arms as well as to the shape of the pit. Therefore, we could not use the position of the limbs as a clear indicator of wrapping. Fortunately, the preservation of the ribcage and the position of the beads strengthened our interpretation that the infant had been wrapped for her burial. Duday *et al*. (1990) mention that a naked ribcage covered by sediment will not collapse, as the pockets liberated by decomposing soft tissue will gradually be filled by sediment. The 3D location of the human remains and beads reconstructed via photogrammetry shows that this is not what we see at Arma Veirana; the distal ends of the ribs are only 1 cm above their proximal ends, suggesting that the ribcage collapsed in as the body decomposed. Combined with our interpretation that the pit was filled with sediment, this suggests that the infant was wrapped in fur, hide, or even textile, which took longer to decompose than the body itself. Unfortunately, we did not find traces that could help us determine which material was used for the wrap. In the absence of such evidence and given the probable use of textile as early as the Upper Paleolithic (*e.g.*, Adovasio *et al*., 1996; Soffer *et al*., 2000), we here refer to this piece as a *wrap*.

The 3D placement of all aspects of the burial (bones, shells, charcoal, and ochre) shows a slight concentration of ochre just above where the abdomen would have been. While we are cautious about calling it a line as it consists only of 5 small specimens (< 1 cm^3^), the number of ochre pieces that were big enough to be collected is rare within the fill sediment, which makes this alignment noteworthy (see S11 in Hodgkins *et al*., 2021). Moreover, the abdomen and head shells have more ochre than the shells found on the arm or feet. The 3D position of the ornaments studied here also suggests that they had been sewn on the wrap. Below, we analyze the in situ arrangements linked to different body parts separately (cranium, arm, and abdomen) to detail this interpretation.

#### Head

**Fig. 20:**
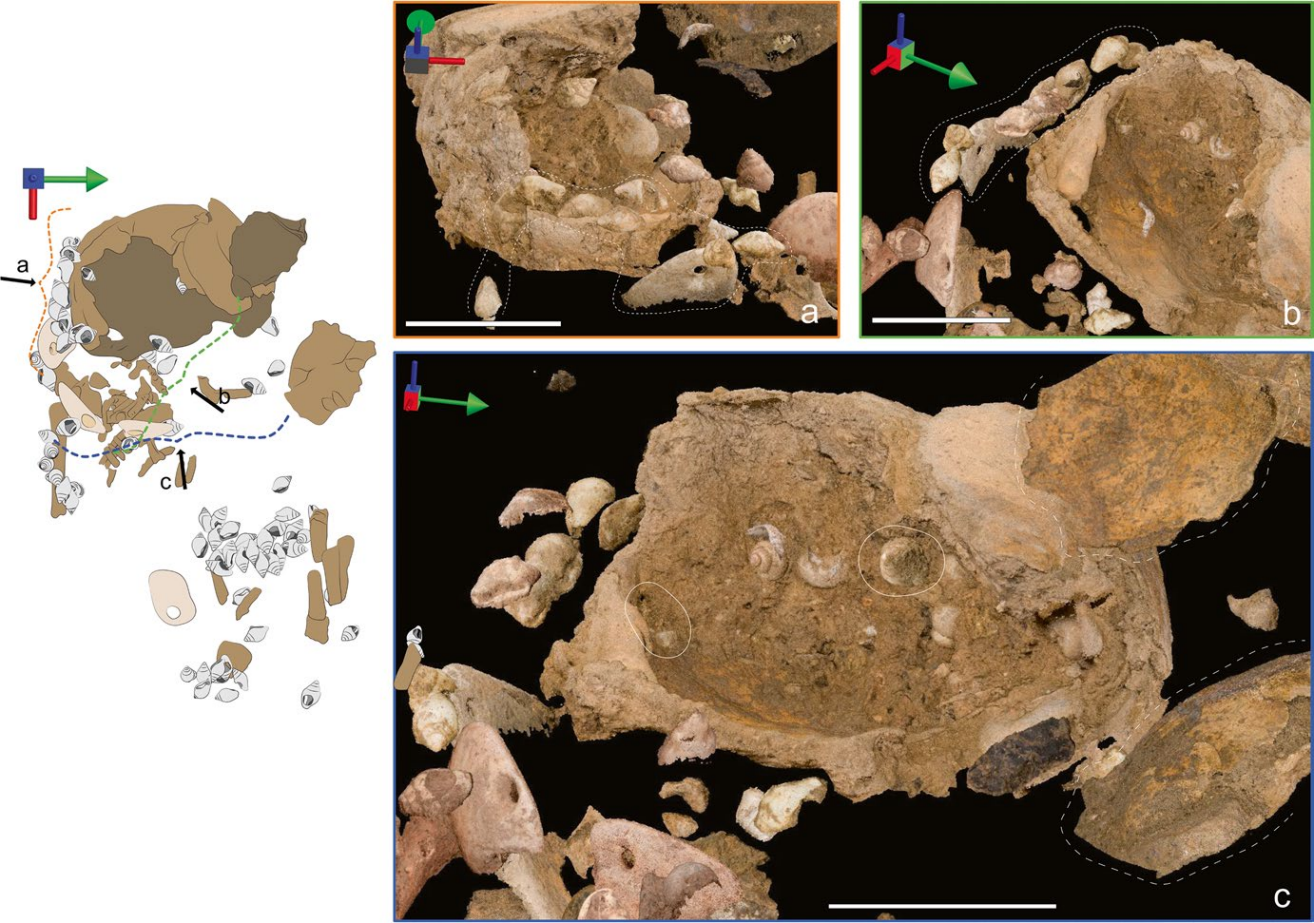
Parallel-projection orthographic views of artifacts and human remains from the photogrammetry model reconstructed from field photos. The white bar measures 4 cm for all images, and the multidirectional arrow shows the viewpoint of the image, where the green arrow points to north, the red to east, and the blue points up. The drawing on the left can be used as a reference to identify the location of each image. The dotted curly brackets show the viewing angle of the scans viewed from the side. **A** and **B** The dotted line shows some of the perforated columbellae and one pendant found aligned right next to the cranium. Note that the unperforated half of the pendant is not shown as it was under the cranium and thus could not be captured by the field photos. **C** Different viewpoints of the cranium and perforated columbellae. The white lines show the position of two perforated columbellae found inside the cranium, whereas the hatched lines show the position of two cranium fragments that had been disturbed post-interment

Twelve perforated columbellae were found immediately adjacent to the right parietal cranial piece, in a line that followed the curve of the cranium and extended underneath it (see Figs. 16 and 20a and b). The 3D position of the preserved shell line led us to believe that the beads were not part of a piece of jewelry deposited on top of the buried infant but were rather sewn on the wrap holding the infant. This interpretation is supported by the three shells found underneath the cranium which may have been part of the same bead line, as well as two perforated *C. rustica* found inside the cranium itself (Fig. 20c). Given that unfused pieces of the cranium were detached and moved aside by post-depositional disturbances (see Figs. 3 and 20c), it is possible that those two shells, which had originally been on top of the cranium, fell inside it when the bones were disturbed.

**Fig. 21:**
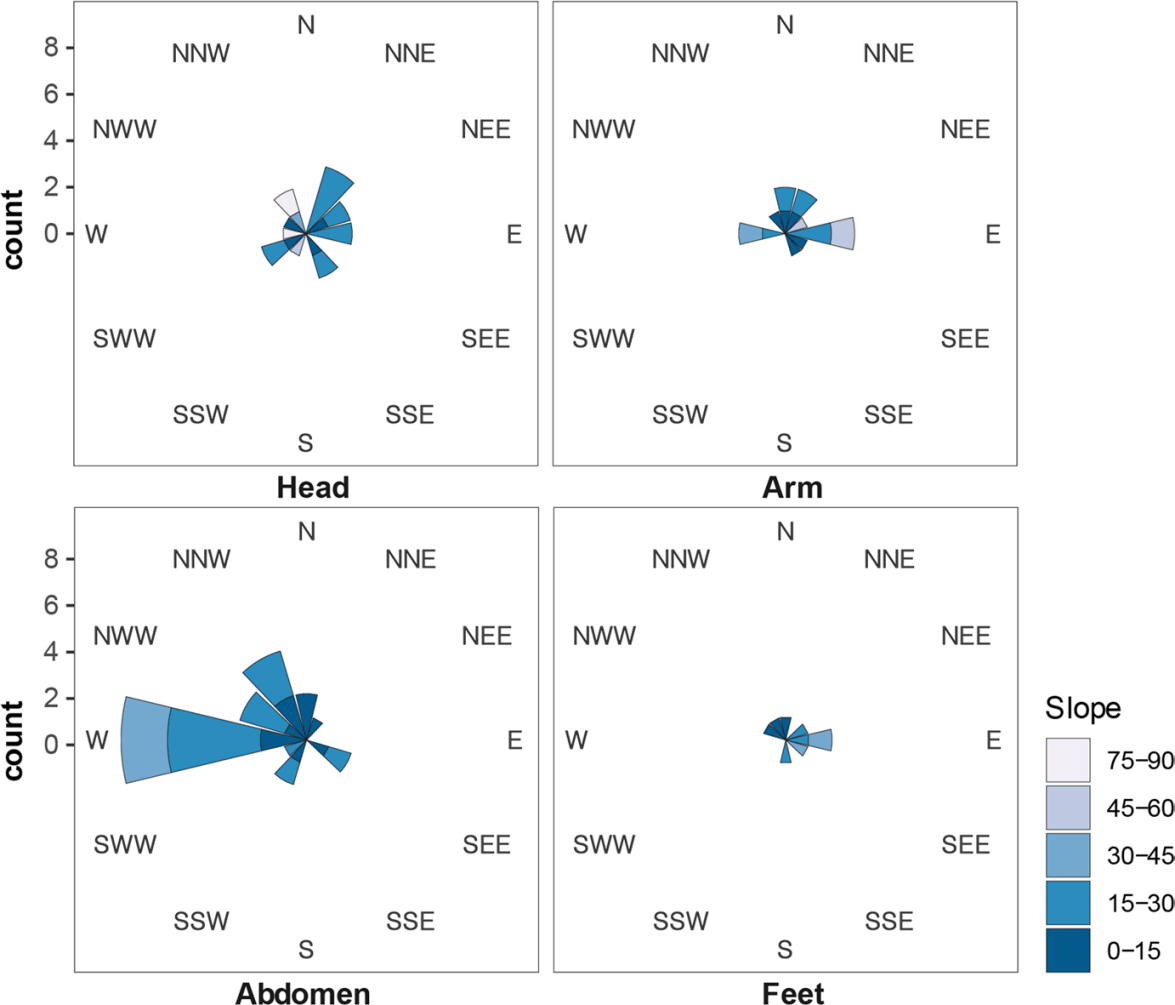
Slope and direction of perforated shells mapped by the total station. The length of each portion represents the count of shells that were found in the direction and slope represented by the cardinal direction and color, respectively

Most of the shells found to the right of the cranium were found aligned one above the other, with their opercula facing the perforation of the neighboring shell—with one exception where both perforations faced one another. Most of these shells followed a slope of 15–30° oriented in almost all directions (Fig. 21).

Pendant #9228 was found at a pronounced angle directly abutting the right portion of the cranium with its perforated half higher than its unperforated half. The 3D reconstruction of the burial shows that this pendant was likely part of the head arrangement, as its perforation aligns well with the perforation and opercula of two surrounding columbellas.

#### Arm

The eight shells found on top of the arm formed a line curving around the right humerus and toward the torso (Fig. 19a and d). Most of these shells were found tightly packed with their top spires pointing up. All shells were placed with their opercula facing the perforation of their neighboring shell. We can be sure that these shell beads were accompanied by pendant #3854 as its perforation was adjacent to the opening of one *C. rustica*. It is highly possible that the line of shells found above the arm continued onto the torso, where pendant #3858 and two more *C. rustica* were found. All those shells were located above the bones, suggesting that they were sewn on the wrap used to bury the infant or that they were part of an ornament deposited on top of the body.

**Fig. 22:**
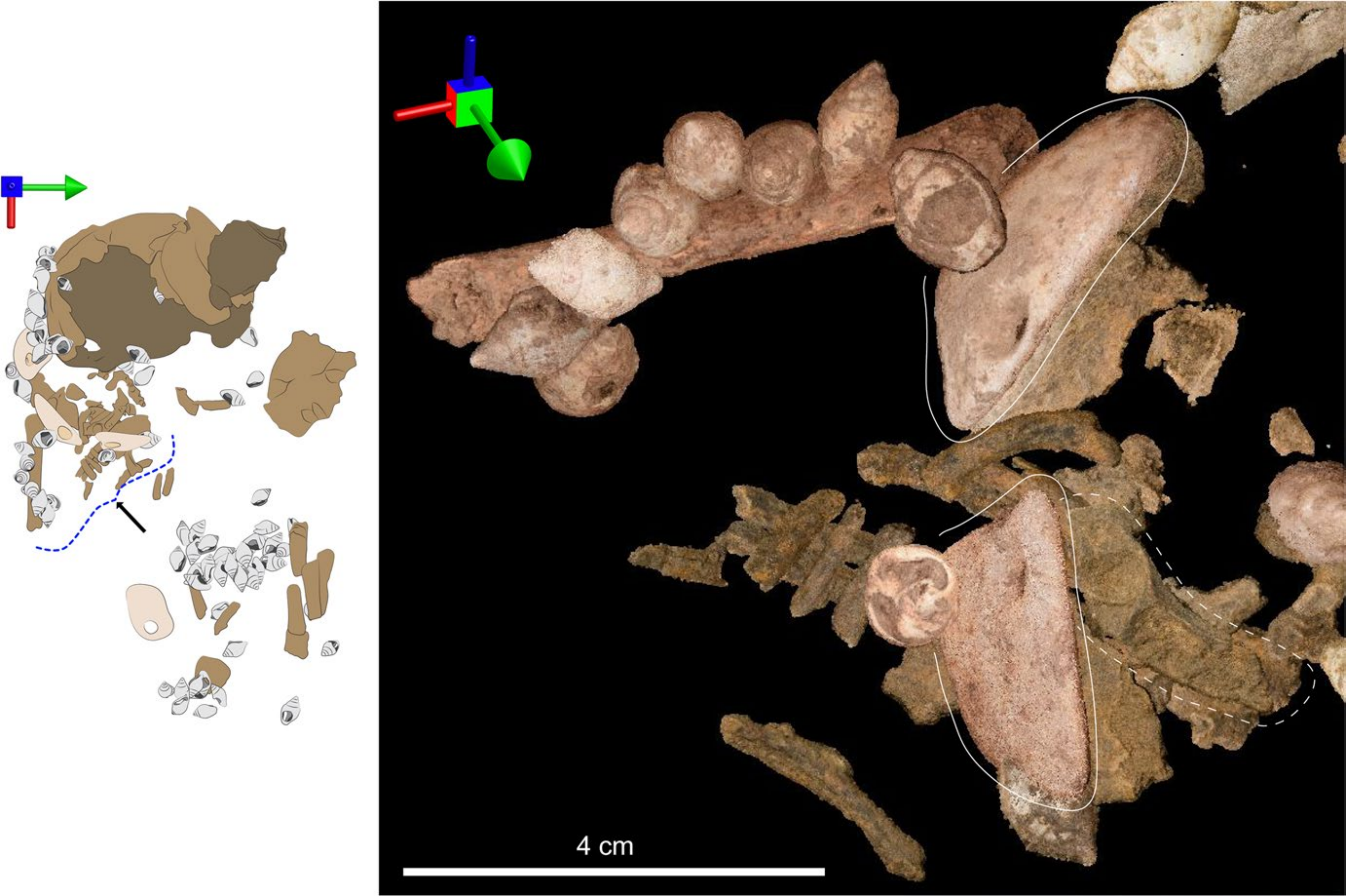
Parallel-projection orthographic views of artifacts and human remains from the photogrammetry model reconstructed from field photos. The multidirectional arrow shows the viewpoint of the image, where the green arrow points to north, the red to east, and the blue points up. The drawing on the left can be used as a reference to identify the location of the image. The dotted curly brackets show the viewing angle of the scan viewed from the side. This image shows the proximity of the two pendants (full white line) and the left hemi-mandible (dotted line)

The two pendants (3854 and 3858) were found with their ventral side almost abutting the left hemi-mandible (Fig. 22). At the time of discovery, they were standing almost vertically, following the same slope as the underlying ribs (lower toward the East), which suggests that their excavated position resulted from post-depositional displacement following the ribcage collapse. Therefore, at the time of burial, the pendants were most probably lying flat with their ventral side against the torso of the infant and with their perforation facing one another. As they remained above the ribs, it is more than likely that the pendants were sewn on the wrap that covered the body.

#### Abdomen

**Fig. 23:**
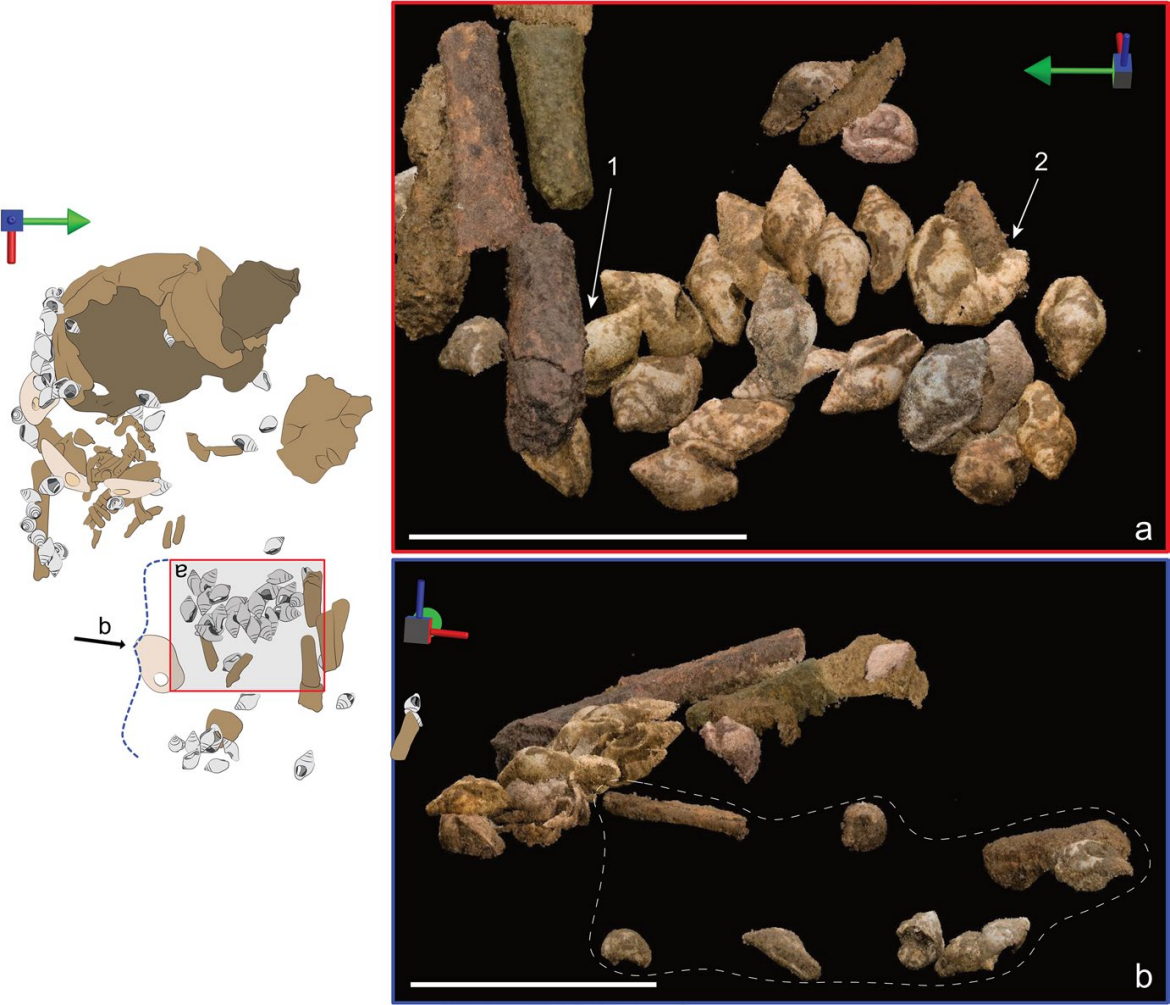
Parallel-projection orthographic views of artifacts and human remains from the photogrammetry model reconstructed from field photos. The white bar measures 4 cm for all images and the multidirectional arrow shows the viewpoint of the image, where the green arrow points to north, the red to east, and the blue points up. The drawing on the left can be used as a reference to identify the location of each image. The rectangle relates to a view of the scan seen from above, whereas the dotted curly bracket shows the viewing angle of the scan viewed from the side. **A** The relative position of the perforated columbellae found in the abdomen area. The arrows show the long bones that were found above (1) and below (2) the beads. **B** Side view of the beads found in the abdomen area. This shows the sloped angle toward the head as well as the beads and bones that were pulled down into a burrow (dotted line)

The arrangement found where the abdomen would have been contains 22 shells. 3D placement suggests that the abdomen shells were arranged in two lines, with the one closest to the head being deeper than the other (Fig. 23). Most shells found in this region follow a ~ 15–30° slope tilted toward the west (Fig. 21). Incidentally, this is the same slope as the long bones they accompany. Archaeothanatology indicates that as the stomach decomposes, it bloats and then collapses, which should lead to the collapse of any grave goods deposited over it (as discussed in Haglund & Sorg, 2002, p. 104). This explains why the shells found above the abdomen are sloping down toward the head; however, it does not explain why the shells retained their relative alignment. If the beads had been deposited on top of the abdomen as a piece of jewelry, they likely would have lost their relative connections during the collapse of the stomach. Therefore, given that most of those shells remained in a tight alignment, the most parsimonious explanation is that they were sewn on the wrap, which sloped down “as one piece” as the body decomposed.

Understanding the position of this arrangement in relation to the body is difficult. The 3D position of the two lines shows that they were slightly higher than the ribs, which support our interpretation that the beads were originally above the body rather than below it. However, none of the lumbar vertebrae were found during the excavation or in the sieved material, probably due to post-depositional disturbances (see SI in Hodgkins *et al*., 2021) as well as decomposition of fragile infant bones. Moreover, one isolated long bone fragment (probably belonging to the right lower limb) was found below the higher line of shells, while a cluster of other long bone fragments (likely belonging to the left lower limb) were found immediately above shells from the same line (Fig. 23a). This presents a conundrum as the line of shells could not have been simultaneously both above and below the lower limbs. However, the long bone fragment that likely belonged to the right lower limb is aligned with bones and artifacts excavated from sediment that has a different composition than the rest of the burial fill, suggesting the presence of a burrow. Therefore, the right long bone may have been in the process of being pulled down into the burrow. On the other hand, the long bones of the left lower limb were clustered together in lines that appear mostly undisturbed (apart from regular taphonomical displacement). Therefore, those probably represent better the original position of the lower limbs. With this in mind and the probable wrapping discussed earlier, it is logical to think that the beads found in this arrangement were placed between the abdomen and the folded legs when the infant was buried.

Similar to the arm and head arrangement, most of the shells found on the abdomen were placed with their opercula facing their neighbor’s perforation. However, here, we see more instances of shells placed with their perforation facing one another. As the shells were tightly aligned, it is likely that they were attached to one another rather than individually secured to the wrap, as the latter method would have left larger spaces between shells. Moreover, the position of the two shell lines leaves the possibility that those may have been connected at one or both extremities, perhaps where they were secured to the wrap.

In the section below, we discuss the use of both perforated *C. rustica* and *Glycymeris* pendants within the broader archaeological context.

## Discussion

Our detailed analysis provides new information on ornaments and the ways in which they were likely used for prehistoric infants. This multi-disciplinary research combined a wide array of methods (GIS, micro-CT, photogrammetry, use-wear and residue analysis, experiments) to provide as much information as possible about an extremely rare discovery. To ensure the robustness of our analyses, we compared the results obtained from different methods, such as our visual analyses of the GIS data and the photogrammetry model. This allowed us to ascertain the accuracy of our interpretations of the relative position of ornaments and human remains. We also calculated the angles and the roughness around the perforations of *C. rustica* to complement our use-wear analyses. Interestingly, the results of the roughness and summed angle analyses aligned well with the results of our qualitative microscopic analyses, which demonstrate the reliability of the optical assessment for measuring use-wear intensity. In addition, the computation of average roughness allowed us to explore potential correlations between it and other values such as the size of the perforation area, as others have hypothesized that bigger perforations might indicate longer use (Taborin, 1993). Here, the statistically significant negative correlation between roughness and perforation size supports this hypothesis along with previous research that noticed a similar pattern (Perlès, 2018). This suggests that wider perforation may be a sign of longer wear. Interestingly, average roughness correlated also significantly with thickness, which may be a sign that repeated usage and friction tend to round thicker shells’ perforation edges, whereas it will instead break thinner shells’ edges, which will then remain relatively fresher. This interpretation needs to be explored further using experimentation.

In the discussion below, we first place the ornaments within their social context. Then, we summarize the characteristics of the burial, and finally refer to ethnographic research to support our interpretation of how the beads were used by the infant and its community, in life and in death.

### The Use of Columbella rustica

*Columbella rustica* lives on submerged rocky shores in warm waters and is commonly found along modern Mediterranean shores (Bertolini *et al*., 2016; Cartonnet, 1991; Cristiani, 2012; Taborin, 1993). As Arma Veirana is located < 20 km from the present-day Mediterranean coast, it is possible that the shells were collected locally from the nearest source (*i.e.*, the beach in Albenga); however, further studies are needed to confirm or refute this possibility.

Ornaments made from this species have been found in multiple European sites, spanning the Aurignacian to the Neolithic (*e.g.*, Álvarez-Fernández, 2010; Biagi *et al*., 1987; Taborin, 1993) and it was the most popular taxon for shell ornaments in the Mediterranean region during the Mesolithic (*e.g.*, Cristiani *et al*., 2014; Cvitkušić, 2017; Newell, 1990; Perlès & Vanhaeren, 2010; Stiner, 2014). Columbellae have been found in several Mesolithic sites from the Ebro Valley of the Iberian peninsula (*e.g.*, Álvarez-Fernández, 2006; Martínez-Moreno *et al*., 2010) to the Balkans (*e.g.*, Benghiat *et al*., 2009; Cristiani *et al*., 2014; Cristiani & Borić, 2017), suggesting the presence of a wide-ranging and intricate network of social relationships connecting those southern European regions. In fact, here we argue that the groups who buried the infant at Arma Veirana were likely part of this social network, as the columbellae found in the burial conform to Early Mesolithic ornamental norms through their taxonomy, *chaîne opératoire*, ochre coverage, and use-wear patterns, which are detailed below.

While their spatial distribution is extensive, columbellae are often found in relatively small numbers in Early Mesolithic sites across the Italian peninsula and around the Adriatic Sea (*e.g.*, Álvarez-Fernández, 2006; Mussi, 2001; Taborin, 1993). Only a few sites have yielded more than 40, including Vlakno Cave (*n* = 352), Vela Spila (*n* = 338), and Pupićina Cave (*n* = 94) in Croatia (Cristiani *et al*., 2014; Cvitkušić, 2017), Franchthi cave in Greece (*n* = 141 in the Lower Mesolithic, Perlès, 2018), and Grotta di Pozzo (*n* = 45, Brunelli *et al*., 2016), Grotta Continenza (*n* = 73, Colombo & Serradimigni, 2015), Romagnano Loc III (*n* = 76, Borrello & Dalmeri, 2004), and Grotta della Serratura (*n* ~ 500, Mussi, 2001). The Croatian sites of Vlakno and Vela Spila also have yielded many unperforated columbellae and about half of the shells found at Serratura were unperforated, suggesting that the beach near those sites may have been an important source for this raw material for ornament making. Therefore, while the people of Arma Veirana may have collected their shells within Liguria, it is also possible that they did so through some connections with people living in the Adriatic or the Tyrrhenian region. Future research will help us answer this question.

The average size of modern columbellae ranges between 11 and 14 mm in length, depending on the source. The size of the specimen found at Veirana falls on the higher part of that range, a pattern that has been observed at other sites (*e.g.* Perlès, 2018). The bigger size of the shells used for the burial can be explained by the fact that bigger shells are easier to perforate (Perlès, 2018). However, it should be noted that the fact that those shells, buried with an infant, are not smaller than specimens found in other archaeological assemblages, which suggests that they were not a “child” version of adult ornaments, as hypothesized at other children burials (*e.g.*, Vanhaeren & d’Errico, 2001; White, 1999).

When used as ornaments, columbellae are usually perforated on the side opposite their natural opening. Perforations are often large—although size varies per site—and quadrangular or subcircular in shape with straight edges or inwardly expanded cone fractures (*e.g.*, Benghiat *et al*., 2009; Dalmeri & Fiocchi, 1998). The upper areas of the perforations are sometimes chipped, which has been identified as a likely result of using direct percussion to create the perforation (Bertolini *et al*., 2016; Cristiani *et al*., 2014, 2020). The Arma Veirana assemblage fits this description perfectly.

Direct percussion is a perforation method commonly associated with *C. rustica* (*e.g.*, Álvarez-Fernández, 2006; Benghiat *et al*., 2009; Micheli, 2004; Stiner *et al*., 2013; Perlès, 2018), although some have hypothesized that indirect percussion could have been an alternative technique used to perforate such shells (Cristiani *et al*., 2014, 2020; Perlès, 2018). Because most shells from Arma Veirana are worn on the top part of the perforation, this prevented us from identifying the required diagnostic crushing on a large portion of the ornaments. To remedy this, we are currently performing more systematic experiments to confirm our interpretation of the analyzed assemblage.

The shells found in the Arma Veirana burial exhibit only minimal ochre staining and do not show black staining, which is common in Early Mesolithic burials (Grünberg, 2016). At Arma Veirana, those low levels suggest that the shells themselves were likely not colored. Instead, we suggest that one of the following alternative explanations best explain the origin of the ochre: 1) the ochre specks transferred onto the shells from the decorated wrap or an ochred string; 2) the ochre traces are remnants of the arrangements they were used in prior to being sewn on the wrap; or 3) only a select few shells were colored to create a striking visual contrast between beads of different colors. Modern *C. rustica* found on the Mediterranean shore are naturally brown to red or gray (Pauc & Pauc, 2006), but shoreline activity bleaches the shells over time, leading to white specimens (*e.g.*, Benghiat *et al*., 2009; Perlès, 2018). Most of the Arma Veirana columbellae show signs that they were collected from thanatocenosis on the beach (sponge and bioerosion marks); therefore, some of them may have been white at the time of collection. While a few archaeological specimens still show a faint outline of their original colors, it is possible that some specimens were selected for their color or that bleached shells were subsequently recolored. However, it does not appear that all the burial shells were intentionally colored red, given that coloring the shells would have left more residues within the spires than what has been identified in the assemblage.

The type and location of the use-wear documented on Arma Veirana columbellae also fit the pattern found in other archaeological assemblages. At most Mesolithic sites, perforated *C. rustica* are well worn, with rounding found especially at the top of their perforation and on the inside part of their outer lip (*e.g.*, Bertolini *et al*., 2016; Cristiani & Borić, 2017; Perlès, 2018), which is concordant with what is seen in the Veirana assemblage. The long use life of *C. rustica* beads seen at most sites was only possible because of the shells’ durability, which may have been one of the reasons why they were selected in the first place (see Stiner, 2014). Some have hypothesized that the shells were attached through the lips so that the ventral part would show (Bertolini *et al*., 2016; Cristiani, 2012), but our 3D reconstruction of the burial shows that the visible part of the shells might instead have been the side between the natural and anthropogenic openings, a pattern also identified in the Neolithic burial of Avignon (Zemour *et al*., 2017).

### The Glycymeris Pendants

While the columbellae found in the burial fit well within the ornamental norms of the Early Mesolithic documented above, the fit of the four Glycymeris perforated pendants found in this burial remains mysterious, as similar ornaments are extremely rare in the European prehistoric record. *Glycymeris* shells have been very popular throughout prehistory; they were found in Middle Paleolithic sites (Zilhão *et al*., 2010) as well as Middle-Eastern and African Middle Stone Age sites more than 90,000 years old (Bar-Yosef Mayer *et al*., 2009; Vanhaeren *et al*., 2013). However, in almost all archaeological instances, *Glycymeris* shells were used as complete valves, sometimes strung through their perforated umbo or the distal part of their valves were used as half-moon pendants, likely attached using a string around their middle (*e.g.*, Álvarez-Fernández, 2010; Anfossi, 1972; Bar-Yosef Mayer *et al*., 2009; Borić & Cristiani, 2019; Borrello, 2004; Micheli, 2004; Peresani *et al*., 2019; Rivière, 1887; Tripković *et al*., 2016; Vanhaeren & d’Errico, 2011). In European sites, up to recently, single *Glycymeris* perforations were always found on the umbo (Taborin, 1993, p. 284).

An extensive literature review shows that perforated pendants with a shape similar to the ones found at Arma Veirana have been found further West in Liguria at the Balzi Rossi (Mussi, 2001), in the Late Epigravettian in northeastern Italy at Riparo Tagliente (Accorsi Benini, 1972; Cilli *et al*., 2006) and Riparo Dalmeri (Borrello & Dalmeri, 2004), and in the Epipaleolithic of l’Aven des Iboussière in France (d’Errico & Vanhaeren, 2000); however, in those cases, the pendants were made on bone or stone rather than on shell. Perforated *Glycymeris* fragments with a similar shape have only been found in a handful of contexts: the Late Epigravettian of Grotta del Romito—here, as a pendant in making as the perforation was never finished (Martini *et al*., 2007)—the Capsian of the Grotte du Polygone, in Algeria (Cornaggia & Girod, 1965), and in the Bronze Age Sardinian occupations at Padru Jossu (Borrello, 2004) and Anghelu Ruju (Puddu, 2014). However, all those specimens are either older or much younger than those recovered at Arma Veirana, thus preventing us from linking them directly to the group who buried the infant.

While the shape of the Arma Veirana pendants is unique for their date, ornaments made on half-moon-shaped *Glycymeris* fragments have been found in association with Late Epigravettian burials in the nearby site of Arene Candide (~ 35 km from Arma Veirana). However, these pendants differed in their method of suspension; they were hung with a string wrapped around their middle (Cardini, 1980), whereas the Arma Veirana pieces were all hung through their perforation. Moreover, the pieces found at Arene Candide were thinner than the ones found in direct association with the burial, which suggests that the Veirana pieces came from bigger shells, despite being buried with a younger individual, an interesting pattern that is discussed below. The pieces found at Arene Candide—including the > 200 still unpublished specimens mentioned by Cardini (1946)—are characterized as semi-lunate fragments. Similar semi-lunates have been documented in multiple Late Epigravettian sites throughout southern Europe and Northern Africa (Cornaggia & Girod, 1965), which suggests a certain level of connectivity between all those locations. However, without other contemporaneous similar specimens, it is difficult to understand how the Arma Veirana pendants fit within the Early Mesolithic cultural context. To this date, they are certainly one of a kind.

As mentioned in the introduction, personal ornaments are thought to communicate identity, gender, or status (*e.g.*, Cristiani *et al*., 2014; Cvitkušić, 2017; Kuhn & Stiner, 2007), as well as to help maintain social relationships (Wiessner, 1982), and protect from evil (Miller, 2009). In the perspective of beads as a communication device, the taxa combination seen in the assemblage studied here suggests that the group who buried the infant maintained strong ties with the highly connected population of Early Mesolithic southern Europe (*C. rustica*), while keeping a certain individuality (*Glycymeris* sp.).

### Reconstruction of the Burial Process

The analysis of the perforated shells combined with the taphonomy of the burial suggests that the infant was wrapped in a piece of textile, fur, or hide that was decorated with several arrangements of perforated shells and pendants. The wrapped infant was deposited in a small pit with her legs folded over her abdomen and was covered with sediment.

The 3D position of the beads found near the abdomen suggests that they were placed between the abdomen and folded legs. Combined with our interpretation that the infant was wrapped, it is possible that those shells were sown on the wrap itself or on a piece of clothing such as undergarments, which was then covered by the wrap. Unfortunately, we cannot identify which of those possibilities is the correct one. However, we can assume that, by folding the legs of the infant over the abdomen, the group who buried the infant likely hid some of those ornaments, thus removing the beads’ aesthetics from the burial. This suggests that the beads were not buried with the infant to serve as funerary decoration, but were rather part of a decorated garment or baby sling that was likely used during the infant’s life (see Miller, 2009), similarly to other burials of children that have yielded abundant ornaments (Boric *et al*., 2014; Cardini, 1980; Cristiani & Borić, 2017; Henry-Gambier, 2001; Vanhaeren & d’Errico, 2001). This is different from the burial of the infants at Krems-Wachtberg, who were interred with beads that did not bear any use-wear and were thus likely made specifically to serve as grave goods for the burial (Teschler-Nicola *et al*., 2020).

**Fig. 24:**
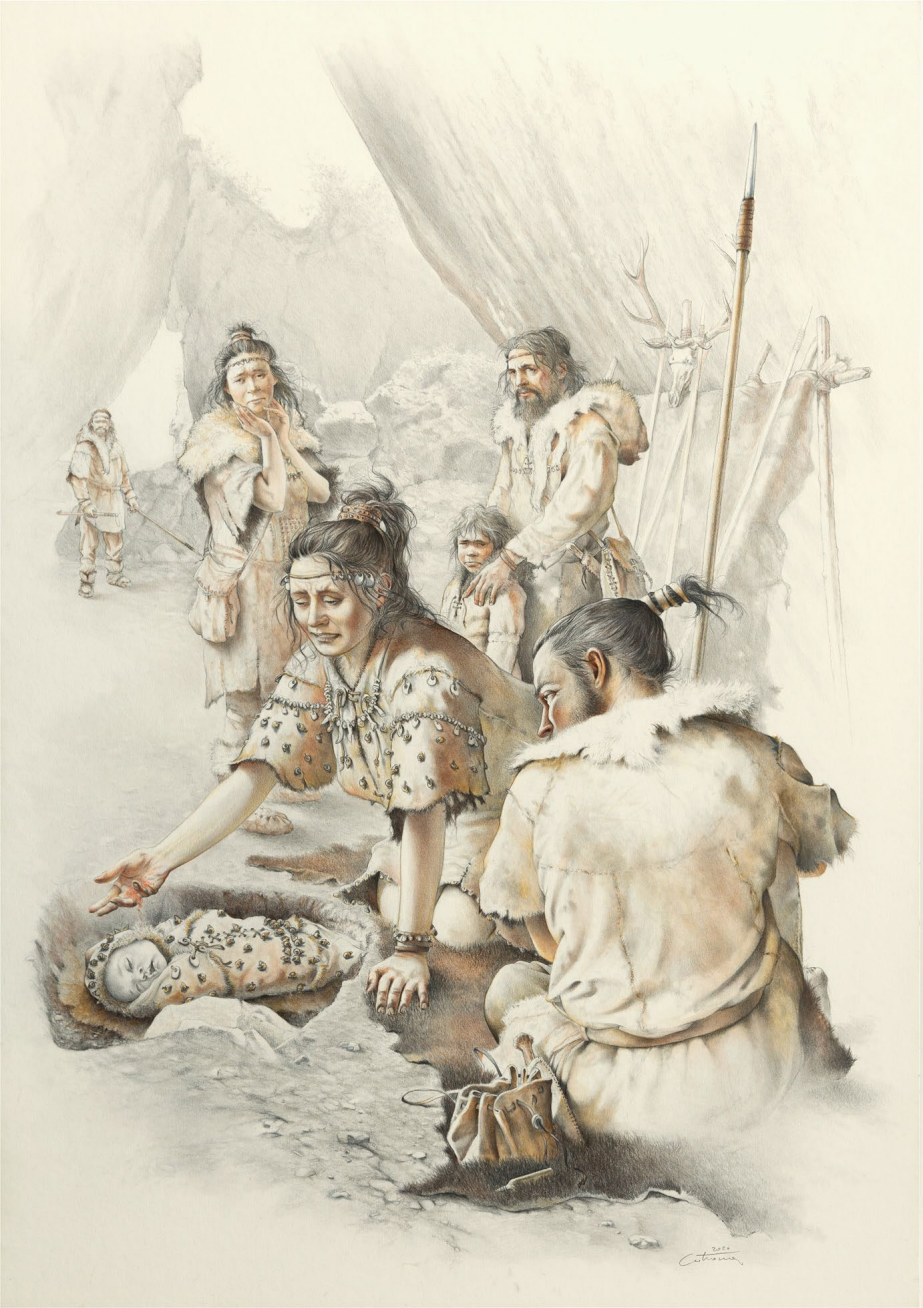
Artistic reconstruction of the Arma Veirana burial (drawing by Mauro Cutrona)

The 3D position of the beads curved around the humerus and next to the cranium suggests strongly that the shells were sewn on the wrap used to bury the infant. Most of those beads were aligned tightly close together, which suggest that the shells were likely strung together and the string was then sewn on the wrap. Furthermore, the widespread location of ochre residues on several shells suggests that the strings used or the wrap itself were likely treated with some ochre, which transferred to both the internal and external parts of the shells, as inferred for other archaeological assemblages (Rigaud, 2011; Velliky *et al*., 2018). Ochre was found in higher quantities on shells located near the head and the abdomen, which suggests that ochre may have been preferentially applied near the head and the abdomen as shown in the artistic reconstruction of this burial (Fig. 24). However, as the human remains were not tinted red nor the sediment around them, it is unlikely that big quantities of ochre were applied to those regions during the burial. Alternatively, the higher level of ochre found on shells located near the head could result from a different type of stringing and ochre arrangement or from their longer usage as they were significantly more worn than the ones found near other body parts. It is possible that those beads accumulated ochre throughout their lengthy use in different arrangements when worn by other people.

This brings us to our interpretation that most ornaments found in the burial had been used for an extended period of time by the group who buried the infant before the infant was even born. This interpretation is supported by the extensive use-wear recorded on most shells, the numerous rounded fractures found on the siphon canal of columbellae, and the location of facets on a few of them.

Vanhaeren and d’Errico (2011, p. 69) have argued that the intensity of use-wear traces on ornaments can be an indicator of how long they were used. In the Arma Veirana burial, the extensive degree of wear and low surface roughness measured on the columbellas could not have been produced during the short life of the buried infant (40–50 days). Therefore, the level of wear probably developed while the beads were used over time by other members of the community before being inherited by the infant during her short life.

This interpretation is supported by the location of worn facets found on a few columbellae, which were likely created through friction against another hard surface (Fig. 16). While friction between shells can produce such facets in other taxa (Vanhaeren *et al*., 2013), the position in which we found the facetted shells suggests that the facets were created before the beads were “given” to the infant, as they are located on surfaces that would not have been in contact with other shells. While we cannot reject the possibility that some hard objects made on organic matter were part of the arrangement when the infant was alive and did not preserve in the burial, the most parsimonious interpretation is that the facets were created while the shells were worn in different arrangements by other members of the group/family prior to being sewn on the wrap in which the infant was buried.

In addition to the facets and use-wear, the presence of numerous rounded fractures in the assemblage of columbellae suggests that the shells were used for an extended period of time after the fracture occurred. The sources of the fractures are unknown; they could be the result of the previous use of the ornaments in specific arrangements involving compression between shells or they could simply be collateral damage incurred during perforation. However, such fractures are relatively rare in contemporaneous assemblages of columbellae, which suggests that they were likely not produced by natural processes prior to being collected.

The pendants as well were likely worn by the community for a considerable amount of time before they were given to the infant. The location of use-wear on those pendants suggests that they were worn with their ventral side against the body, which is consistent with how they were found in the burial. This suggests a certain continuity in how those pendants were worn by the group and by the infant. Therefore, it is possible that the shells transferred to the baby in life or death may have acted as a link between her and her close relatives, a possibility we discuss further below.

Microscopic analyses show that the Arma Veirana pendants were collected as broken fragments on the beach. We cannot prove that the foragers who collected those fragments were looking for a specific taxon; however, it is possible that they were selecting for a specific color, shape, finish, smoothness, and thickness, as all pendants are roughly similar in size and finish. This in itself points to the symbolic aspect of these pendants, which means that people might have overlooked utilitarian concerns—thinner fragments would have been easier to perforate—in search for a certain subjective beauty or meaning. While most Arma Veirana pendants have natural red coloring on their ventral side, it is unlikely that they were selected for that characteristic because the use-wear patterns show that the ventral side was hidden from view. Instead, the pendants were possibly selected for their white dorsal color, especially as the low amount of ochre residues found on those fragments suggests that they were not intentionally colored red. In fact, the ochre traces found on the pendants are so sparse that it suggests that they could have come from an ochred string used to bind a previous arrangement or simply from contact with a few ochre specks in the sediment.

Excavation around the burial pit uncovered an eagle owl talon that had briefly been used as an ornament and left in a small pit adjacent to the burial, which was interpreted as an offering (Hodgkins *et al*., 2021). Pendant #3484 was found at the same elevation as the eagle owl talon (~ 6 cm directly above the burial) and may have been another offering placed there intentionally (as per Arias’s (2012) definition). The reasoning behind this interpretation is the following: (1) Identical pendants have not been found at any contemporaneous sites in the region and beyond, as we discussed above. Therefore, the coincidence of having one pendant directly above a buried individual interred with three identical pendants makes it very unlikely that the 4th pendant was unrelated to the three buried ones or that it was lost there by mistake. (2) This pendant was found immediately adjacent to the only large rock (> 10 cm in length) documented above the remains. Rocks of similar size or bigger were found at all elevations around the burial pit, suggesting that they are natural occurrences in the cave. However, they are completely absent from the 6 cm of sediment found immediately above the human remains (see S1 video). The fact that the pendant was found adjacent to a big rock and at the same elevation as the eagle-owl talon, therefore, suggests that the pendant was deposited on top of the burial fill after the funeral, and that it was then covered by natural accumulation of small roofspall and sediment over time. (3) This pendant was more altered than the three recovered in the pit, which could have been due to a longer exposure to atmospheric conditions that produce a characteristic white patina on certain bivalves (Manca, 2016). (4) We did not find signs of soil disturbance around the 4th pendant nor the land snails that were found in high quantities in the sections of the burial that had been disturbed by taphonomical processes (*e.g.*, near and inside the cranium and around the ribs). All these combined suggest that the pendant was deposited intentionally on top of the covered burial. Interestingly, if our interpretation is correct, this would suggest that the burial remained undisturbed for a relatively long time, which may even suggest that the cave remained unoccupied during that time. More research is needed to confirm this interpretation, however.

Finally, the placement of the ornaments on the Arma Veirana infant body is interesting as it is focused on the region around the head and the abdomen, where shells also have more ochre traces than near other body parts. The head and abdomen are two body parts that are often decorated with ornaments in prehistoric burials. This is especially true for the abdomen/pelvis region in children, as seen in the children burials of Krems-Wachtberg, Grotta dei Fanciulli, Sungir, Bogebakken, and Vlasac (*e.g.*, Cristiani *et al*., 2014; Cristiani & Borić, 2017; Grünberg, 2016; Henry-Gambier, 1995; Riel-Salvatore & Gravel-Miguel, 2013; Vang Petersen, 2016). As different body parts have different symbolic meanings in modern forager societies (Vanhaeren & d’Errico, 2011), this may suggest that these two lines were purposely sewn above the abdomen for a symbolic purpose such as to protect and strengthen their vital body parts (as seen in Miller, 2009). On the other hand, decorated headwear are common in Upper Paleolithic burials of individuals of all ages. Those have been interpreted as implements to broadcast social information, due to the high visibility of the head when viewed from a distance (Riel-Salvatore & Gravel-Miguel, 2013). Combined with the intense use-wear of the shells found near AVH-1’s head, this suggests that the shells used to convey social belonging may have been intentionally reused for as long as possible to retain and strengthen their symbolized social identity.

Therefore, AVH-1 was likely interred with ornaments composed of beads that had already been used by her community. Combined with our analysis, which showed that shells with very different levels of wear were found within single arrangements, this suggests that perforated shells were recycled on a regular basis and remixed into new arrangements. In addition, the fact that shells placed near the head are significantly more worn than others suggests that certain shells were specially selected to adorn that body part and thus may have had a special significance. In the next section, we refer to ethnographic research to explain why we believe that the wrap on which the beads were sewn was likely used as a baby sling while the infant was alive.

### Exploring Community Intentions Surrounding the Burial

The high number of beads found in the AVH-1 burial is impressive. Up until recently, this would have been seen as a marker of high status. However, one ethno-anthropological research into the role of material culture in Indigenous societies reveals that in some modern Amazonian societies, forager groups perceive body decorations and ornaments as materializations of the parental care toward a child (Miller, 2009). During the first years of life, such care are crucial for the health of the child and in this context, ornaments represent a reflection and an extension of this care, and protection from evil. Not surprisingly, in those societies, infants and children are always well adorned. Among the beads that are used to decorate and protect their bodies, the majority are “second-hand” items, *i.e.*, beads that have been donated by the parents, grandparents, and relatives as an act of care toward the child. Accordingly, ornaments play a key role in “building” children’s bodies through social relationships and protection from diseases. They hence become material evidence of the network of relationships linking the child to the members of the community, which are necessary for the child to become “human.”

In certain modern forager populations, such decorations are placed on baby carriers and slings (Vang Petersen, 2016). As archaeologists are increasingly discussing the possibility that baby carriers and slings were widely used in prehistory (Langley & Suddendorf, 2020; Nowell & Kurki, 2020; Suddendorf *et al*., 2020; Taylor, 2010), re-analyses of infant burials have hypothesized that some infants were, in fact, buried in such carriers. For example, grave 7 of La Vergne contains the remains of two adults next to the remains of a child that were placed in a box decorated with multiple perforated marine shells and animal teeth (Laporte & Dupont, 2019; Laporte *et al*., 2021) that could be interpreted as a carrier. Recent re-analysis of the infant of Abri Labattut provides another example, as this research shows that the infant was buried with multiple cowrie shell ornaments that were too big (~ 2.5 cm in length) to have been worn as jewelry or on the clothes of such a small individual, and which use-wear suggests that they had been attached to a fixed object, possibly a blanket, baby carrier, or other (Henry-Gambier *et al*., 2019, p. 199). Coincidentally, this is reminiscent of the Arma Veirana infant, who was buried with three *Glycymeris* pendants that were as big as the cowries of Labattut (mean ~ 3.3 cm in length vs. ~ 3.2 cm for Labattut) and were associated with an even younger individual (45–50 days old vs. < 10 months old for Labattut). As we tried to understand how the pendants would have fit on a piece of clothing used for a ~ 2-month-old infant, the possibility that those would have adorned a carrier seemed more logical and practical.

Moreover, as discussed in the introduction, recent research has shown that ornaments were likely used to produce sensorial experiences for the infants (Rainio & Mannermaa, 2014). Artifacts from Mesolithic and Neolithic burials have been interpreted as rattling ornaments (Larsson, 1984, p. 20; Rainio *et al*., 2021; Rainio & Mannermaa, 2014; Rainio & Tamboer, 2018). Based on these, we should not rule out the possibility that the pendants from AVH-1, standing out for their way of suspension, their dimension, and developed traces, might have produced a rhythmic sound when hitting each other.

Going even further, Vang Petersen hypothesizes that the ornaments used on carriers could have symbolized “amulets” used to protect the child. Moreover, he highlights that among the Pueblo Indians of the American Southwest, the carrier of a child who dies is burned with them to avoid passing over the “defective” amulets to someone else (Vang Petersen, 2016, p. 119).

While it is impossible to prove, we hypothesize that a similar scenario could explain the presence and 3D location of the ornaments found in association with the Arma Veirana infant. As mentioned above—and similarly to the documented use-wear on some of the ornaments associated with the child of La Vergne (Laporte *et al*., 2021)—the use-wear documented on the ornaments shows that they had been worn for long periods by members of the community prior to the burial (and indeed the birth) of the infant. Some of those beads could have previously been used for other baby carriers for infants who survived to adulthood and thus, whose ornaments could be passed on to the new baby girl. However, as she did not survive, it may have been deemed safer to bury her with her adorned carrier than to reuse the ornaments that failed to protect her in life. We believe that, given the ethnographic and archaeological examples detailed above, this hypothesis is possible.

Alternately —although, not mutually exclusive—the presence of the pendants in the burial could have also embodied the long-lasting connection to and care from parents, relatives and community members to the child. This could explain why the group decided to part with beads that had been curated for a considerable amount of time rather than use new shells they could have obtained from the nearby coast. In fact, this strongly suggests that the beads found here were more than just decorations, as shells used only as burial embellishment would not have needed to be durable (Benghiat *et al*., 2009). Finally, another possibility is that the adults intentionally parted with their own ornaments to adorn a ceremonial shroud, providing a link between the dead and the living, as was probably done in the Late Epigravettian of the Arene Candide with intentionally broken pebbles used as spatulas to decorate the deceased with ochre as part of larger funerary rituals (Gravel-Miguel *et al*., 2017). Distinguishing between all these possible interpretations will require more research.

## Conclusion

In summary, our study shows that most ornaments discarded in this burial were worn for longer than the lifespan of the interred infant. This indicates that the beads were recycled or passed between individuals of different ages within the group and certain beads were likely selected specifically to adorn special parts of the body. The burial taphonomy suggests that the baby was buried in a decorated wrap placed carefully within the small burial pit. The wrap may have been used as a sling in which the baby was kept close to her parents in the few days she was alive. The beads used to decorate it may have been viewed as protective “amulets” that were discarded with the baby when she died to avoid reusing ornaments that had “failed” or may have symbolized the connection between the group and the deceased individual. The use of *C. rustica* shells conforms with the norm of the Early Mesolithic, while adding a certain individuality through the use of unique *Glycymeris* pendants. This suggests that the community who buried the infant was an integral part of the well-connected Early Mesolithic southern European social network.

## Supplementary Information

Below is the link to the electronic supplementary material.

Supplementary file 1 (MOV 161023 kb)

Supplementary file 2 (CSV 15 kb)

Supplementary file 3 (RMD 24 kb)

Supplementary file 4 (PNG 549 kb)

High resolution image (TIF 1817 kb)

Supplementary file 5 (PNG 747 kb)

High resolution image (TIF 4055 kb)

Supplementary file 6 (PDF 317 kb)
