# Supplementary figures and images for: The Ornaments of the Arma Veirana Early Mesolithic Infant Burial
Source: J Archaeol Method Theory. 2022 Aug 30;30(3):757–804. doi: 10.1007/s10816-022-09573-7 (PMC10432373; doi:10.1007/s10816-022-09573-7)

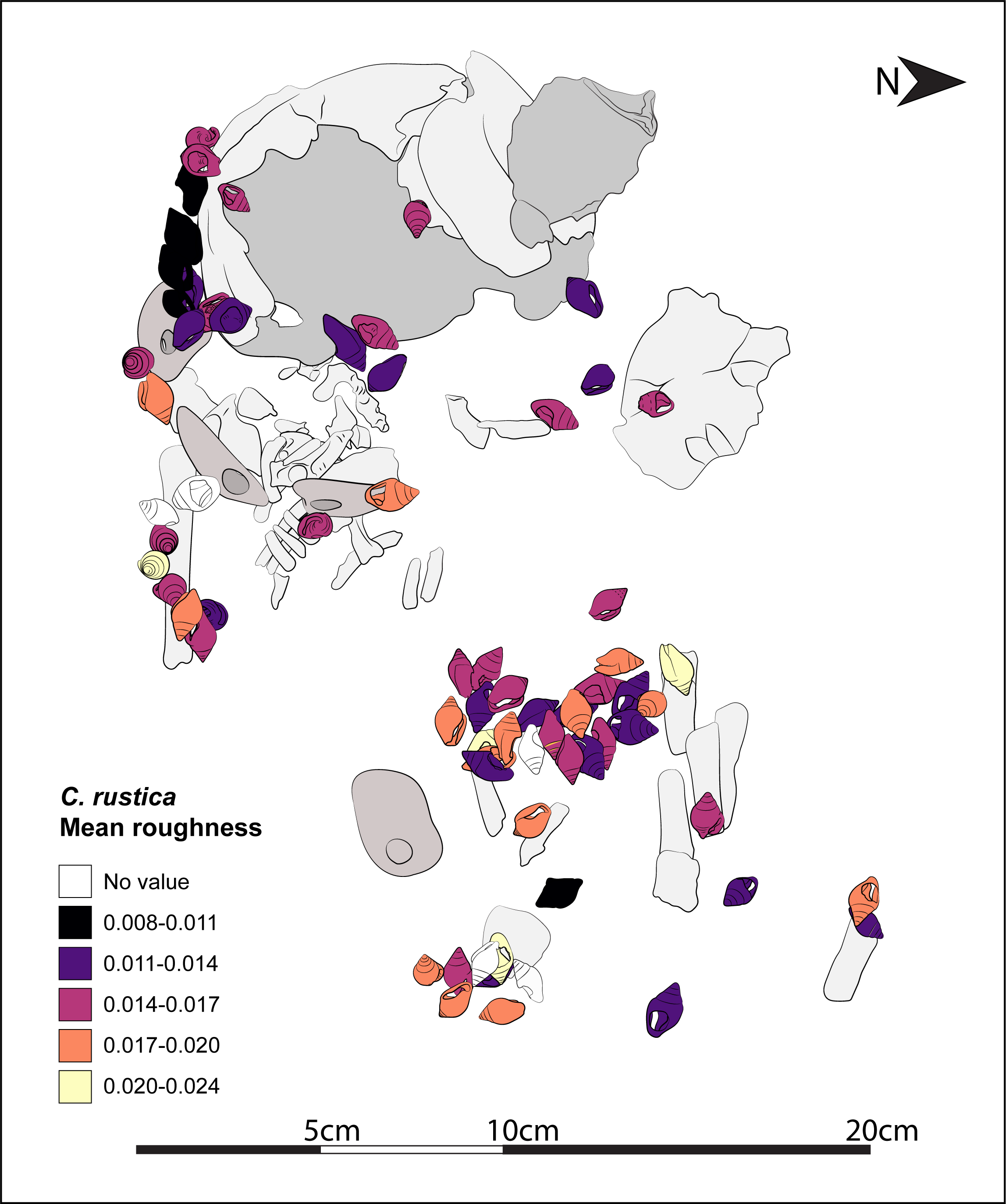

Supplement: Supplementary file 4 — (PNG 549 kb) [file 10816_2022_9573_Fig25_ESM.png]

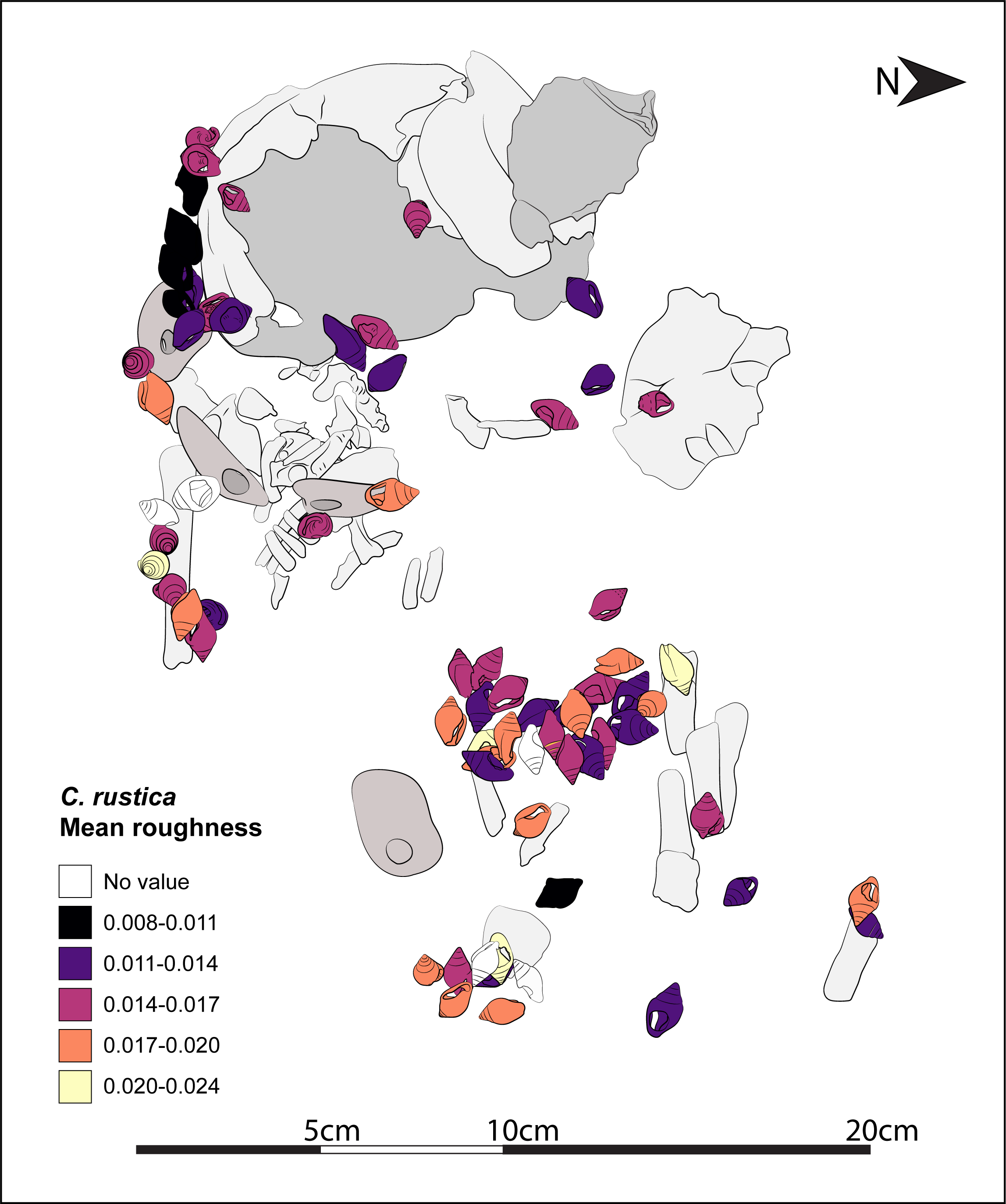

Supplement: Supplementary file 5 — High resolution image (TIF 1817 kb) [file 10816_2022_9573_MOESM4_ESM.tif]

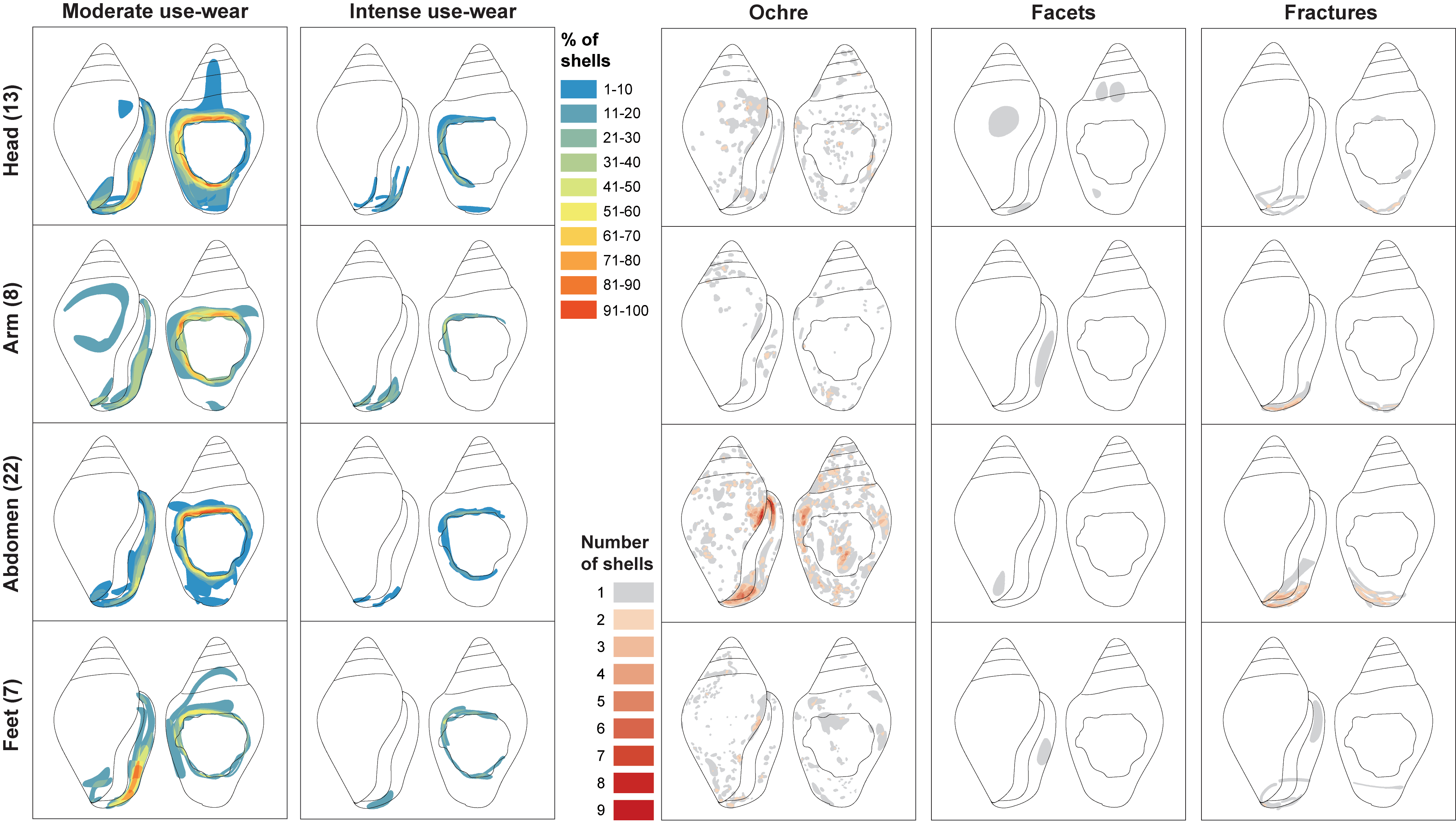

Supplement: Supplementary file 6 — (PNG 747 kb) [file 10816_2022_9573_Fig26_ESM.png]

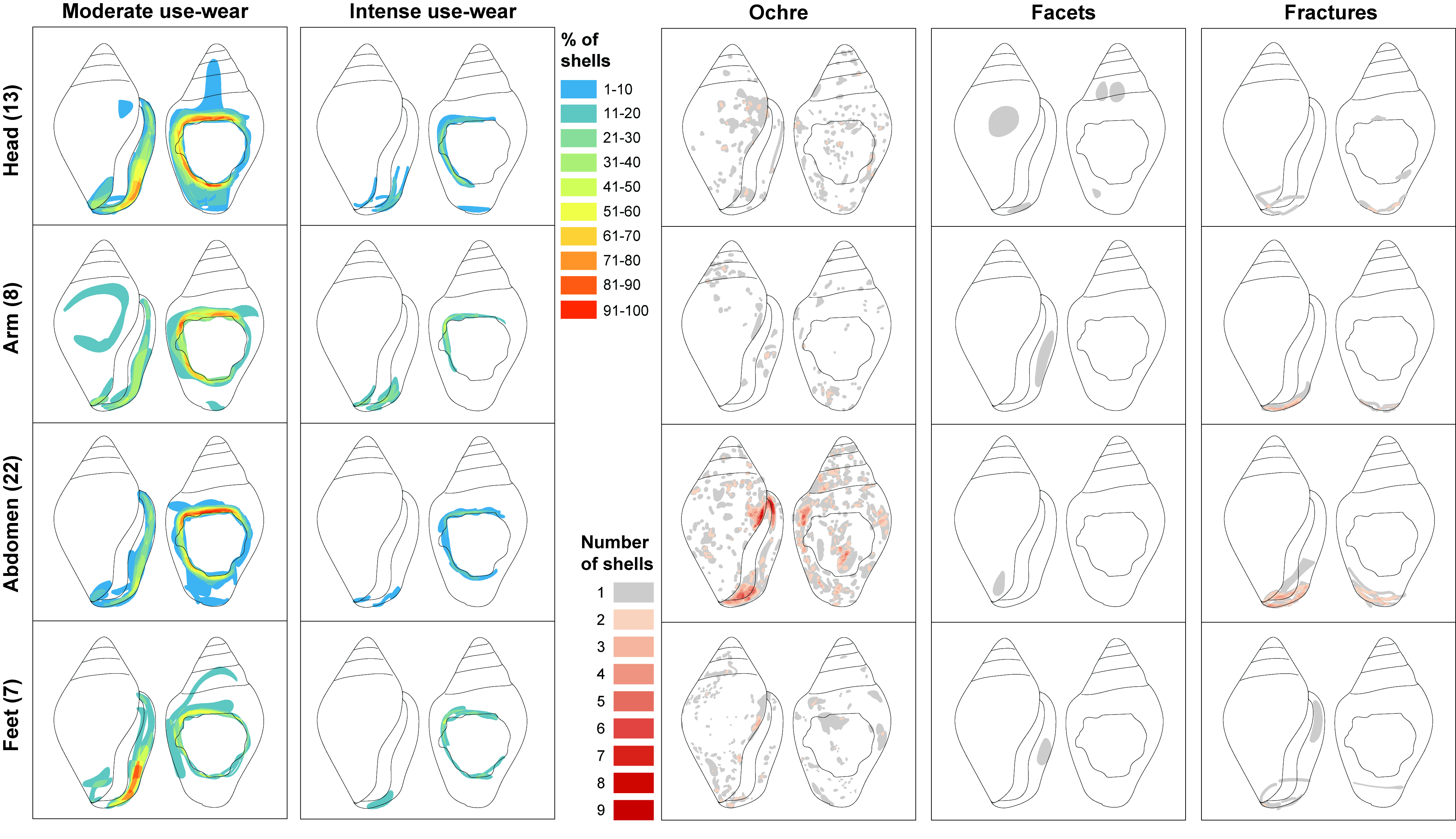

Supplement: Supplementary file 7 — High resolution image (TIF 4055 kb) [file 10816_2022_9573_MOESM5_ESM.tif]
