# Supplementary material for: The Ornaments of the Arma Veirana Early Mesolithic Infant Burial
Source: J Archaeol Method Theory. 2022 Aug 30;30(3):757–804. doi: 10.1007/s10816-022-09573-7 (PMC10432373; doi:10.1007/s10816-022-09573-7)
Supplement: Supplementary file 8 — (PDF 317 kb) [file 10816_2022_9573_MOESM6_ESM.pdf]

Supplementary Information

S1 File. Video reconstructing the depth of each artifact, human remains, and rocks documented in the burial. The video starts from the bottom and goes up by 1cm increments. As it moves up, the artifacts that are in full color are those with a base depth between the shown depth and the cm above it. The artifacts that are 50% transparent are those that are in the cm below what is visible at each 1cm increment.

S2 Table. Dataset of shells' measurements, geographical coordinates, and use-wear scores. The variables are the following (*refers to variables only applicable to *C. rustica*). A visual aid for the different measurements is available below.

- PF: Plotted find number, which indicates if the shell was found in sieved sediment (NA) or mapped using the total station (#).
- Lot: Number attributed to layers in individual quads. A shell can have a lot# even if it does not have a PF# because sieved sediment is separated by each quad and is thus linked to its lot#.
- X: Latitude
- Y: Longitude
- Z: Depth
- Slope: Slope in degree of the shell. This measure is available only for shells longer than they are wide and that were clear components of the burial. For those, we took a GIS coordinate on both extremities, and calculated the slope between the two. If the shell was not longer than wide (or if it was excavated before we realized they were part of a burial), we could not calculate the slope (NA).
- Orientation: The cardinal direction towards which the shell slopes down, calculated using the same extremity coordinates as the slope.
- Location: Closest category of human remain, based on 3D distanced between the shell and all the human remains plotted with the Total Station.
- Group: Subjective binary classification that identifies if the shell was part of an in-situ cluster (line of shells).
- Taxon: Identified taxon of each shell.
- Length: Measure of the longest part of the shell (mm).
- Width: Measure of the widest part of the shell, perpendicular to the length (mm).
- AreaBodyWhorl*: Area of the body whorl (cm^2).
- LengthBodyWhorl*: Measure of the body whorl between the dorsal and ventral sides (cm).
- PerfLength: Measure of the perforation size, in the direction following the length of the whole shell (cm).
- PerfWidth: Measure of the perforation size, in the direction following the width of the whole shell (cm).

- AreaPerf: Area of the perforation (cm^2).
- RatioBodyFull*: Percentage of the shell length covered by the body whorl (%).
- RatioAreaPerf*: Percentage of the body whorl area covered by the perforation (%).
- Thickness: Thickness of the shell, at the right side of the perforation.
- UseWearDorsal*: Use wear score of the 4 dorsal locations around the perforation. Range of possible values: 0-12.
- UseWearVentral*: Use wear score of the 3 ventral locations around the natural opening. Range of possible values: 0-9.
- Note: notes explaining lack of values for some shells. E.g., shell #3077 was selected for dating before we could observe it under the microscope and take measurements.

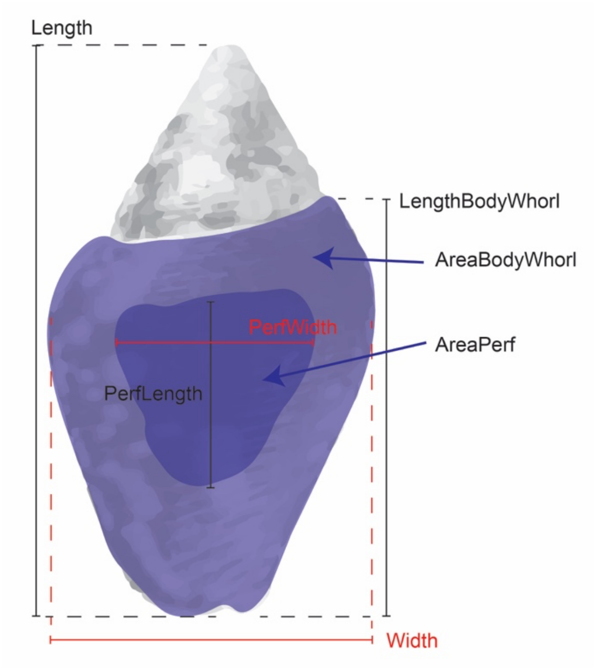

S3 File. R script used to analyze the shells' morphology and use wear score.

S4 Fig. Spatial distribution of the *C. rustica* colored by use-wear score.

S5 Fig. Use-wear, ochre, facets, and fractures spatial distribution divided by groups based on their location on the body. The color scale represents % of shells for use-wear, and the number of shells for ochre, facets, and fractures.
